# Enrichment of different taxa of the enigmatic candidate phyla radiation bacteria using a novel picolitre droplet technique

**DOI:** 10.1093/ismeco/ycae080

**Published:** 2024-06-21

**Authors:** DeDe Kwun Wai Man, Syrie M Hermans, Martin Taubert, Sarahi L Garcia, Sundar Hengoju, Kirsten Küsel, Miriam A Rosenbaum

**Affiliations:** Leibniz Institute for Natural Product Research and Infection Biology – Hans Knöll Institute (HKI), 07745 Jena, Germany; Balance of the Microverse, Cluster of Excellence, Friedrich Schiller University, 07743 Jena, Germany; Balance of the Microverse, Cluster of Excellence, Friedrich Schiller University, 07743 Jena, Germany; Food Science and Microbiology, School of Science, Faculty of Health and Environmental Sciences, Auckland University of Technology, 1142 Auckland, New Zealand; Aquatic Geomicrobiology, Institute of Biodiversity, Faculty of Biological Sciences, Friedrich Schiller University, 07743 Jena, Germany; Balance of the Microverse, Cluster of Excellence, Friedrich Schiller University, 07743 Jena, Germany; Aquatic Geomicrobiology, Institute of Biodiversity, Faculty of Biological Sciences, Friedrich Schiller University, 07743 Jena, Germany; Department of Ecology, Environment and Plant Sciences, Science for Life Laboratory, Stockholm University, 106 91 Stockholm, Sweden; Institute for Chemistry and Biology of the Marine Environment (ICBM), School of Mathematics and Science, Carl von Ossietzky Universität Oldenburg, 26129 Oldenburg, Germany; Leibniz Institute for Natural Product Research and Infection Biology – Hans Knöll Institute (HKI), 07745 Jena, Germany; Balance of the Microverse, Cluster of Excellence, Friedrich Schiller University, 07743 Jena, Germany; Aquatic Geomicrobiology, Institute of Biodiversity, Faculty of Biological Sciences, Friedrich Schiller University, 07743 Jena, Germany; German Centre for Integrative Biodiversity Research (iDiv) Halle-Jena-Leipzig, 04103 Leipzig, Germany; Leibniz Institute for Natural Product Research and Infection Biology – Hans Knöll Institute (HKI), 07745 Jena, Germany; Balance of the Microverse, Cluster of Excellence, Friedrich Schiller University, 07743 Jena, Germany; Institute of Microbiology, Faculty of Biological Sciences, Friedrich Schiller University, 07743 Jena, Germany

**Keywords:** droplet microfluidics, groundwater, candidate phyla radiation, uncultured microorganisms, microbial dark matter

## Abstract

The candidate phyla radiation (CPR) represents a distinct monophyletic clade and constitutes a major portion of the tree of life. Extensive efforts have focused on deciphering the functional diversity of its members, primarily using sequencing-based techniques. However, cultivation success remains scarce, presenting a significant challenge, particularly in CPR-dominated groundwater microbiomes characterized by low biomass. Here, we employ an advanced high-throughput droplet microfluidics technique to enrich CPR taxa from groundwater. Utilizing a low-volume filtration approach, we successfully harvested a microbiome resembling the original groundwater microbial community. We assessed CPR enrichment in droplet and aqueous bulk cultivation for 30 days using a novel CPR-specific primer to rapidly track the CPR fraction through the cultivation attempts. The combination of soil extract and microbial-derived necromass provided the most supportive conditions for CPR enrichment. Employing these supplemented conditions, droplet cultivation proved superior to bulk cultivation, resulting in up to a 13-fold CPR enrichment compared to a 1- to 2-fold increase in bulk cultivation. Amplicon sequencing revealed 10 significantly enriched CPR orders. The highest enrichment in CPRs was observed for some unknown members of the Parcubacteria order, *Cand*. Jorgensenbacteria, and unclassified UBA9983. Furthermore, we identified co-enriched putative host taxa, which may guide more targeted CPR isolation approaches in subsequent investigations.

## Introduction

Displaying remarkable prominence in the tree of life, the candidate phyla radiation (CPR) is a superphylum constituting 26% of the diversity of the bacterial domain [1]. It is depicted as a monophyletic branch with massive lineage diversity in the expanded tree of life [2]. Thus, members of CPR are ubiquitous in the microbial world and have been shown to be abundantly present in a broad range of habitats, such as groundwater [2–5], lakes [6–8], geysers [9, 10], soil [11–13], ocean [14], and the human oral cavity [15–17]. They are characterized by an ultrasmall cell size, reduced genomes with sizes below 1 Mbp [18], and limited or not well-known biosynthetic and metabolic capacities. Moreover, some are reported to possess a microaerobic, anaerobic, or fermentative lifestyle [1, 19, 20]. A habitat that seems largely dominated by CPR is groundwater, where CPR reach abundances of 20%–60% [21, 22]. The groundwater CPR range from 0.1 to 0.3 μm in size [21, 22], with genomes estimated to range from 0.59 to 2.8 Mbp with an average of 1.37 Mbp [23–25]. Such small genomes lacking central biosynthetic pathways might indicate a symbiotic lifestyle. Symbiotic lifestyles of groundwater CPR have been proposed [2] based on a series of evidence from cultivation and genomic analyses of CPR from diverse environments, including lakes [26, 27] and mammalian microbiomes [15, 16], and based on cryo-TEM images of groundwater [21, 28]. Another study evidenced the symbiotic association of *Cand.* Sonnebornia with a ciliated protist in freshwater [29]. Given these unusual features, the isolation of CPR taxa has proven very challenging. To date, rare successful coculture isolates include human oral *Cand.* Saccharibacteria with their actinobacterial hosts [16, 17, 19] and a member of the *Cand.* Gracilibacteria class alongside its anoxygenic photosynthetic host [30] from a salt-saturated habitat. However, the recovery—even just the harvesting—of viable CPR from groundwater proved very difficult, as CPR members are quickly outcompeted by other fast-growing heterotrophic bacteria when cultivated in the lab. Therefore, until now, the majority of these taxa have been almost exclusively investigated based on metagenomic analyses [23, 31–34]. More research needs to focus on CPR cultivation strategies that enable sustainable and direct studies of the functional biodiversity of these microbes, thereby deciphering their key roles in complex ecosystems.

To address this research gap, we propose the use of an advanced droplet microfluidics technique, where bacteria are confined in miniaturized compartments of picolitre volume. Droplet cultivation allows massive parallelization and ultrahigh-throughput generation (tens of millions of parallel cultivations) of cultures that highly increase the enrichment success of uncultured microbes from diverse habitats, including soil [35, 36], termite gut [37], and the human gut [38, 39]. Using droplet cultivation, the desired stoichiometric inoculation ratio is estimated by Poisson distribution to steer cultivation from a single cell to small consortia [40]. This favours the cultivation of slow growers like CPR by reaching a balance between reducing bacterial competition and incubating CPR in small communities where it may be possible to find the right putative hosts. We therefore hypothesize that cocultivation in droplets renders possible higher CPR enrichment compared to bulk cultivation. In this work, we leverage the well-studied groundwater of the Hainich Critical Zone Exploratory (CZE) in Thuringia, Germany [41], an aquifer monitoring transect spanning from oxic to anoxic conditions that is characterized by diverse, well-specific microbiomes [42]. Located in the upper aquifer assemblage, the groundwater in well H52 is dominated by CPR with an average relative abundance of >60% of which most CPR are previously undescribed [34]. We devised a cultivation workflow, including steps of microbial harvesting from groundwater, picolitre droplet encapsulation, and incubation with different supplements, and followed this workflow with imaging and genomic analysis of microbial communities. Using this integrated workflow, we were able to enrich specific groundwater CPR taxa in droplets for the first time and demonstrated the powerful utility of this droplet cultivation approach for culturing a wide range of previously uncultured CPR taxa.

## Materials and methods

### Groundwater sampling and microbial cell harvesting

Groundwater was collected at the Hainich CZE [41] along a hillslope transect in Central Germany in December 2021 and January 2022. Ten litres of groundwater sample were collected from the anoxic well H52 (65 m below ground level, mean: 9.6°C) [43] in autoclaved 10-l FLPE (fluorinated polyethylene) containers using submersible sampling pumps (MP1, Grundfos, Denmark). Liquid sterile-filtered Reasoner’s 2A (R2A) media (Sigma-Aldrich), developed to study low growing bacteria from potable water [3, 44], were then added to the groundwater sample at a final concentration of 0.01% and kept in a humidified chamber at 15°C until filtration on the following day.

Groundwater microbial cells were harvested on sterile 0.1 μm Omnipore polytetrafluoroethylene (PTFE) filters (Millipore, Germany) by filtrating 2 l per filter. Twenty millilitres of groundwater filtrate were collected and purged with a filtered gas stream composed of 20% CO_2_ and 80% N_2_ for 30 min. Each filter was sliced into strips and submerged in 3 ml of anoxic groundwater filtrate supplemented with 0.5% Tween 80 (Serva, Germany). The filter strips were then vortexed for 3 h at 4°C for microbial cell detachment. The filters were discarded, and the cell suspension (inoculum) was kept anoxic at 15°C until droplet generation.

### Microfluidic operation and droplet generation

Microfluidic chip design and fabrication were previously published by Tovar *et al.* [45]. Novec 7500 (3M, Germany) supplemented with 2% PicoSurf (Sphere Fluidics, UK) was purged with filtered N_2_ for 30 min before using as the continuous phase. The aqueous phase was formulated with 0.5% Tween 80, inoculum diluted with anoxic groundwater filtrate at a mean occupancy of λ_CPR_ = 5, and different nutrient supplements including (i) soil extract only (SE), (ii) *Pseudomonas* sp. 002 necromass only (Pseud), (iii) Anammox coculture necromass only (Amx), (iv) soil extract and *Pseudomonas* sp. 002 necromass (Pseud + SE), (v) soil extract and Anammox coculture necromass (Amx + SE), and (vi) without any supplement (NS). Soil extract accounted for 33% of the final aqueous volume for droplet generation; necromass was added at a ratio of 2000 cells to every CPR cell. The two phases were actuated by pressure pumps (ElveFlow, France) directed through the PTFE tubing (0.5 mm ID, Chromophor Technology, Germany) into the microfluidic chip. Microfluidic operation was conducted with an inverted microscope (Axiovert 200M, Zeiss, Germany) and imaged in flow with a Basler aca1920-155uc camera (Basler AG, Germany). Microfluidic droplets were generated with a flow-focusing chip where the continuous and aqueous phases intersect at the nozzle, forming water-in-oil droplets at 1500 Hz with a volume of 150 pl. This aerobic droplet generation process took ~0.5 h. Droplet cultivation samples were collected in 4-ml glass vials secured with silicone/PTFE septum-screw caps (Shimadzu, Japan). Each vial contained ~1.3 × 10^6^ droplets. Droplet populations were generated in triplicates and compared with three aqueous bulk cultivations. Aqueous bulk cultivation samples were prepared by pipetting the same formulated aqueous phase into glass vials. Sampling for Day 0 was performed immediately upon sample generation. The head space of the sample containing vials was purged with N_2_ for 20 min and incubated in an anaerobic pot at 15°C. After incubating for 20 and 30 days, sampling of droplets and bulk cultivation was performed. All samples were kept at −20°C until deoxyribonucleic acid (DNA) extraction.

### DNA extraction and quantitative PCR

With the abundance of bacterial 16S rRNA genes in the inoculum, samples from Days 0, 20, and 30 were determined by quantitative PCR (qPCR). Genomic DNA was extracted from all samples using the DNeasy Ultraclean Microbial Kit (Qiagen, Germany) and kept at −20°C until the qPCR assay. qPCR was performed on a CFX Connect Real/Time PCR Detection System (Bio-Rad, USA) using Brilliant II SYBR Green qPCR Mastermix (Agilent Technologies). qPCR was conducted using the primer combinations Bac8Fmod/Bac338Rabc and 684F-CPR/907R (Table 1). Cycling conditions for bacterial 16S rDNA were as follows: initial denaturation at 95°C for 10 min, 45 cycles of denaturation at 95°C for 30 s, annealing at 55°C for 30 s, and elongation at 72°C for 25 s. For CPR-specific PCR, the conditions were initial denaturation at 95°C for 10 min, 45 cycles of denaturation at 95°C for 30 s, annealing at 51°C for 30 s, and elongation at 72°C for 25 s. Standard curves were constructed using mixtures of plasmids with cloned CPR genes and a cloned bacterial 16S rRNA gene. Standard curves were linear from 5 × 10^7^ to 5 × 10^1^ copies, with *R^2^* values of higher than 0.99 and PCR efficiencies ranging from 80% to 99%. All standards were run in triplicate, and all samples were run in replicates (Cq SD < 0.35) [46]. The specificity of the products was confirmed by melting curve analysis.

**Table 1:** Characterization of primers for groundwater microbial community analysis.

Primer	Sequence (5′-3′)	Target	Analysis	Reference
Bac8Fmod	AGA GTT TGA TYM TGG CTC AG	Bacteria 16S rRNA	qPCR	[49, 50]
Bac338Babc	GCW GCC WCC CGT AGG WGT	Bacteria 16S rRNA	qPCR	[49, 50]
Bakt_0341F	CCT ACG GGN GGC WGC AG	Bacteria 16S rRNA	Illumina sequencing	[51]
Bakt_0785R	GAC TAC HVG GGT ATC TAA TCC	Bacteria 16S rRNA	Illumina sequencing	[51]
684F-CPR	GTA GKR RTR AAA TSC GTT	CPR	qPCR	This study
907R	CCG TCA ATT CMT TTR AGT TT	CPR	qPCR	[52]

### 16S rRNA amplicon sequencing and estimation of CPR abundance

Q5 polymerase (New England Biolabs, Germany) was treated with DNases I (New England Biolabs, Germany) according to the procedure described in Mahler *et al.* [35] to remove the residual bacterial DNA from the enzyme production process. PCR amplification of the bacterial V3–V4 region of the 16S rRNA gene was performed in a 25-μl reaction. Illumina sequencing adapters **TCGTCGGCAGCGTCAGATGTGTATAAGAGACAG** and **GTCTCGTGGGCTCGGAGATGTGTATAAGAGACAG** were attached at the 5′ ends of the forward and reverse primers, respectively. PCR was conducted using primer combinations Bakt_0341F/Bakt_0785R (Table 1). The cycling conditions for 16S rDNA were as follows: initial denaturation at 98°C for 3 min, 40 cycles of denaturation at 98°C for 30 s, annealing at 55°C for 40 s, elongation at 72°C for 1 min, and final extension at 72°C for 5 min. The amplicons were sequenced on the Illumina MiSeq platform with 2 × 300 bp paired-end by IIT Biotech (Bielefeld, Germany). Besides, after verifying the unviability of all supplements on solid medium and analysing the sequencing data, we identified that Pseudomonadaceae exhibited a dominated “ghost” abundance in the data and therefore, we excluded the contribution of this taxa in the adjusted estimated number of bacterial copies (Supplementary Fig. S2C). Amplicon sequencing data are available at NCBI under BioProject accession PRJNA993466. The estimated abundance of each CPR order was calculated by multiplying the relative abundance of each CPR taxon from 16S amplicon sequencing and the total bacterial copies of 16S rRNA gene from qPCR [47, 48].

### Bioinformatics analysis

Analysis of all sequencing data was conducted in R version 4.1.2 [53]. The Divisive Amplicon Denoising Algorithm 2 (DADA2) package [54] was used for quality filtering, denoising, inference of amplicon sequence variants (ASVs) from DNA sequencing data, and chimaera removal. Reads were trimmed off left to remove the primers, then truncated to 270 bp for forward reads or 240 bp for reverse reads. Trimming thresholds were determined by manually inspecting the quality profiles, generated using the “plotQualityProfile” command, of a subset of samples. Further, reads with expected error >2 were removed, and those with quality score ≤2 were truncated. Read numbers per sample prefiltering and post merging are provided in supplementary data (File Supp 8). Denoised reads were merged after using the DADA2 core algorithm to infer ASVs. Chimaeras were removed before constructing the ASV table. The SILVA SSU database version 138 was used for taxonomical assignments of ASVs [55].

### Statistical analysis

The Shannon alpha diversity index for richness analyses was determined using the R package Vegan [56]. The sequencing data of samples collected at the same point were merged and further processed by rarefying, using vegan’s “rarefy_even_dept” command, to 58 292 reads per merged sample (corresponding to the lowest sampling effort). Rarefaction curves (Supplementary Fig. S7) confirmed the validity of this number of reads in representing the majority of diversity in the samples. The seed as set to 711 for all analyses to initialize repeatable random subsampling. The richness between two different cultivation conditions was compared, as well as with the two necromass nutrient supplements. Wilcoxon test was used to access the significant differences. Rarefied data were also used to construct the ASV table as inputs for taxa plot. For the beta-diversity-based analyses, the function metaMDS in R package Vegan was used to cluster bacterial community with a Bray–Curtis dissimilarity matrix based on the two groups explanatory variables: cultivation method (bulk or droplet) and the necromass (Pseud or Amx). The significance of the two explanatory variables were accessed with 999 permutations.

## Results

### Successful recovery of microbes from groundwater and generation of picolitre droplets for cultivation

**Figure 1:**
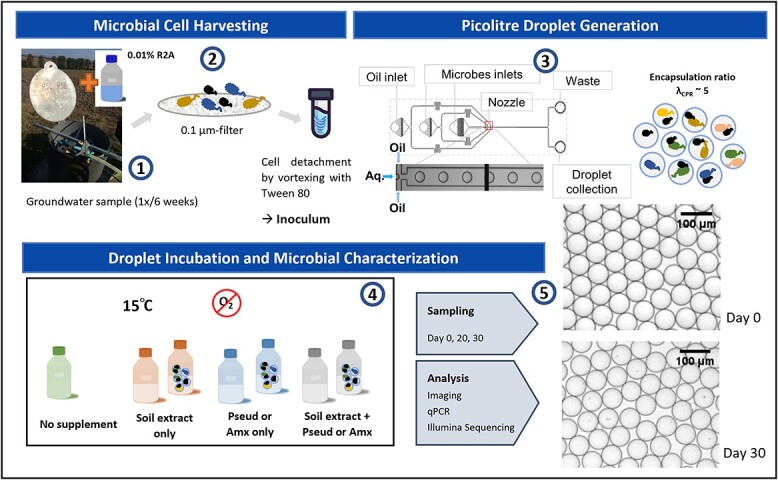
Established workflow of groundwater microbial harvesting and droplet encapsulation of the microbial community [1]. Groundwater samples were collected from well H52 of the Hainich CZE, Germany. Twelve hours before the filtration process, 0.01% R2A was added [2]. Two litres of groundwater were filtered through each 0.1-μm PTFE filter, and cells were detached with Tween 80 to form the inoculum [3]. Droplet generation with a mean occupancy of λ_CPR_ = 5 per droplet was estimated by the Poisson distribution [4]. Droplets and bulk cultivation samples were incubated under anoxic conditions at 15°C with different supplements [5]. Samples were taken on Days 0, 20, and 30 and subjected to downstream analysis. Brightfield microscopy images of droplet samples were taken upon generation on Day 0 and after 30 days of incubation. Pseud and Amx indicate the addition of necromass derived from *Pseudomonas* sp. 002 and an Anammox coculture, respectively.

The CPR cultivation workflow consists of three phases: microbial cell harvesting, picolitre droplet generation, and droplet incubation and microbial characterization (Fig. 1). Groundwater samples were collected at 6-week intervals from the anoxic well H52 located in a depth of 65 m, with a mean water temperature of 9.6°C [43]. Preincubation of the groundwater sample with 0.01% R2A was performed to reduce the starvation-induced cell shrinkage of groundwater microbes [3]. After extensive initial efforts to evaluate different microbial harvesting methods (Supplementary Fig. S1), one-step dead-end filtration of groundwater on a 0.1 μm-PTFE filter, followed by vortexing for cell detachment with the addition of 0.5% Tween 80, was determined as the most efficient method (Fig. 1). This established harvesting protocol rendered the CPR harvested in the inoculum comparable to CPR abundance in the original community [34].

To promote the enrichment of CPR, we mimicked conditions in shallow groundwaters that are characterized by soil surface input. We prepared soil extract (SE) and microbial cells–derived necromass, and applied combinations thereof in our cultivations to resemble the supply of organic matter and of microbial biomass from surface habitats to the subsurface during events such as rainfall and snowmelt. The two types of necromass were prepared by lysing a *Pseudomonas* sp. 002 isolate derived directly from Hainich groundwater (Pseud) and am Anammox coculture derived from a wastewater treatment plant (Amx). Anammox bacteria have been identified at well H52, and CPRs were found to coexist with freshwater Anammox bacteria and possible cometabolic pathways have been predicted using metagenomic analysis [57]. For the generation of ~150-pl droplets, we diluted the inoculum aiming at λ_CPR_ = 5 (on average 5 CPR cells/droplet). This corresponds to an average number of total bacteria of 12 per droplet. Each 2-ml droplet population generated comprised around 1.33 × 10^7^ droplets. Random encapsulation at this ratio could increase the possibility for free CPR to meet the right hosts within the same droplet, while growth competition can be reduced by confining nonhosts in other droplets. To compare the cultivability of CPR taxa in droplets to bulk cultivation, 2 ml of the diluted inoculum with corresponding supplements was placed in glass vials and treated as bulk cultivation. Both droplet and bulk cultivation samples were generated in triplicates. Due to the slower growth and replication rates of groundwater CPR [34], we optimized droplet stability with the addition of biocompatible surfactants in the oil and aqueous phase for long-term incubation. Monodispersed droplets were stable over 4-week incubation at 15°C as verified by brightfield microscopy (Fig. 1).

### Droplet cultivation boosts groundwater CPR enrichment

**Figure 2:**
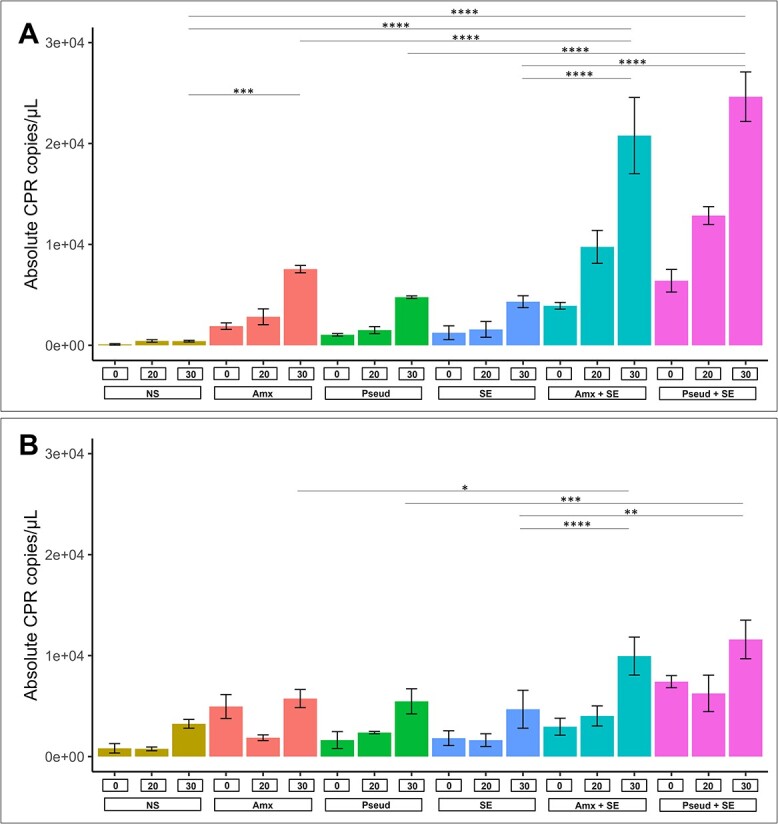
Quantification of CPR subjected to different cultivation conditions. Barplots depict mean and standard deviation of copy numbers of CPR quantified using CPR-specific primer set with qPCR in (A) droplet and (B) bulk cultivation. Groundwater microbial community was subjected to two cultivation conditions (droplet and bulk) with no supplement (“NS”) or the addition of different supplements; Anammox coculture necromass (“Amx”), pseudomonas necromass (“Pseud”), soil extract (“SE”) or a combination of soil extract and necromass (“Amx + SE” “Pseud + SE”). Culture samples were obtained at timepoint day 0, day 20 and day 30; CPR copies in corresponding sample were quantified. Samples were performed in triplicates. Significant difference is indicated between conditions (ANOVA-Tukey test, ^*^^*^^*^ [*P* < .001], ^*^^*^^*^^*^ [*P* < .0001]).

To assess the fate of CPR in our droplet cultivation approach, we employed a novel diagnostic primer to trace the CPR abundance over time and with different nutrient supplements. For this, we pooled the microbial biomass before and after incubation, extracted genomic DNA, and applied quantitative PCR using a newly designed CPR-specific forward primer paired with a universal bacterial reverse primer (validation in Supplementary Information) (Fig. 2).

All conditions led to an increase in the amount of CPR 16S rRNA gene copies through the 30-day incubation. In droplet cultivation, the addition of soil extract to both necromass treatments showed a significant boost of CPR as compared to using only soil extract, Pseud, or Amx alone (Fig. 2A, *P* < .0001). Comparing CPR copy numbers on day 30 to no supplementation (NS), Amx + SE droplets achieved a significant 50-fold higher enrichment at 20 785 ± 4625 (*P* < .0001) copies/μl droplet. Similarly, Pseud + SE droplets also resulted in a significant enrichment at 24 636 ± 3006 copies/μl droplet (*P* < .0001), 60-fold higher than NS. In bulk cultivation, a similar trend was observed with Amx + SE and Pseud + SE supplements achieving the highest CPR enrichment as compared to all other conditions (Fig. 2B). However, this beneficial effect gave a significantly higher CPR enrichment in droplets than in bulk cultivation (*P* < .001), with more than 2-fold difference in CPR copy numbers. Thus, we confirmed an overall superior performance of the highly parallelized droplet cultivation approach compared to classical bulk liquid cultivation.

As the combination of soil extract and necromass showed to be favourable for CPR enrichment in droplets, we repeated these cultivation conditions with the next groundwater sampling. We observed similar trends of increasing CPR copies during the 30-day incubation in all samples with a superior performance of droplet over bulk cultivation (Supplementary Fig. S2) demonstrating the reproducibility of cultivation conditions.

### Deciphering groundwater microbial community enriched in droplets

For a deeper analysis of the cultivability of groundwater microbes in droplet and bulk cultivation, we applied 16S rRNA gene amplicon analyses. Our findings suggest that droplet cultivation addresses a different portion of the groundwater microbiome compared to bulk as detailed in the following.

**Figure 3:**
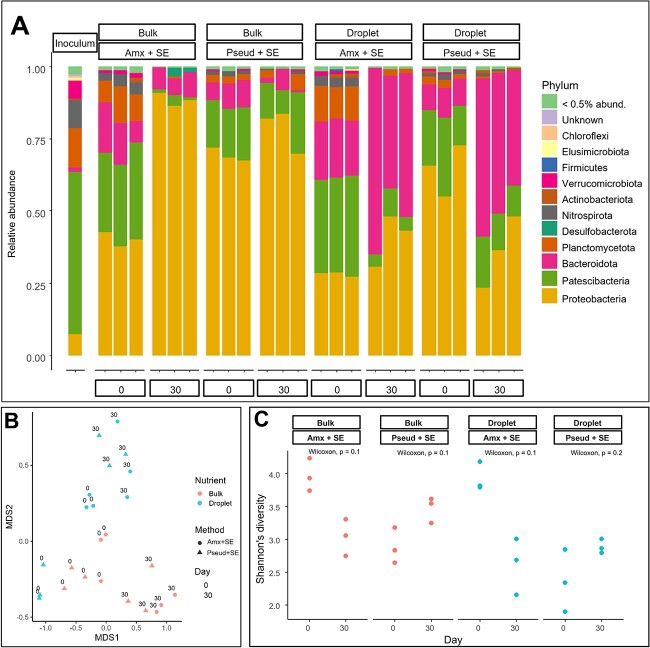
Microbial diversity and community structure of groundwater cultivations. (A) Community structure based on bacterial 16S rRNA gene sequences (relative abundances). Phylogenetic affiliation at the phylum level is shown for each incubation condition. (B) The underlying differences in microbial community composition for samples of two cultivation methods: droplet (cyan) and bulk (coral) with two necromass supplements (shapes) based on Bray–Curtis dissimilarity scores, stress value = 0.1050744. (C) Shannon diversity of groundwater microbiome subjected to two cultivation conditions (droplet and bulk) with the addition of different nutrient supplement (a combination of soil extract and necromass [“Amx + SE” “Pseud + SE”]). Results are presented for the three replicate cultivations; coloured dots represent method of cultivation in corresponding nutrient supplement condition and respective timepoints. Wilcoxon tests were used to assess the differences between groups; *P*-values are showed below the label of each condition.

In total, we detected 679 unique ASVs in our droplet cultivation (Supplementary Fig. S3A). We analysed the bacterial community structure on phylum level between inoculum and different cultivation approaches on day 0 and day 30 (Fig. 3A). Resembling the groundwater community [34], *Cand.* Patescibacteria was the most abundant phylum in the inoculum, accounting for 56.1% of the relative abundance, proving the reliability of our microbial harvesting workflow. Four other major phyla inhabitants were also detected: Planctomycetota at the relative abundance of 13.6%, Nitrospirota at 9.50%, Proteobacteria at 7.41%, and Verrucomicrobiota at 6.23%. It is noticeable that the share of Patescibacteria seemingly decreased to 13.8%–34.8% in preparation of the cultivation samples (D0 for both droplets and bulk). However, this was an effect of dilution at λ_CPR_ = 5 and with the additional necromass, which also contained non-CPR DNA and consequently “diluted” the relative abundance of Patescibacteria. After 30-day incubation, the proportion of Patescibacteria in Pseud + SE remained at 8.50%–21.5% and 10.8%–17.9% in both bulk and droplet cultivation, respectively. Relative abundances of Patescibacteria in Amx + SE were found to be 1.13%–3.83% and 4.32%–9.70% in bulk and droplet cultivation, respectively. Notably, bulk cultivation led to a pronounced promotion of Proteobacteria reaching over 70% in all conditions. In contrast, this phenomenon was not observed in droplets where Proteobacteria remained at 30.8%–48.1% in Amx + SE and 23.3%–48.0% in Pseud + SE. This suggests a positive effect resulting from droplet compartmentalization to restrict certain fast-growing microbial taxa and avoid out-competitions.

To explore the underlying differences in microbial community composition for different cultivation approaches, we calculated the dissimilarity among samples using Bray–Curtis dissimilarity (Fig. 3B). Bacterial community composition differed significantly by nutrient type, cultivation method, and time point (PERMANOVA *P* < .001 for all, Supplementary Table S1). Sampling time explained the greatest portion of variation (*R^2^* = 0.31) followed by method (*R^2^* = 0.17), while nutrient explained the least (*R^2^* = 0.11). Thus, the cultivation methods exerted a decisive effect on day 30 samples leading to a clearly dissimilar microbial community structure in droplets compared to bulk cultivation. On the other hand, the microbial communities in day 30 samples appeared not to be differentiated by the type of applied necromass. There was no significant difference in Shannon diversity between day 0 and day 30 samples in all conditions based on microbial ASVs (Fig. 3C). We cannot rule out that the low number of replicates contributed to the nonsignificant *P*-values. Regardless though, there was a consistent shift in diversity, with Pseud + SE treatments showing an increase and Amx + SE showing a decrease. Comparing samples on day 30, droplets overall resulted in a similar diversity level as bulk cultivation (Supplementary Fig. S4A). Within the total of 1320 ASVs from day 30, droplet cultivation resulted in 38% of unique ASVs as compared to just 18% ASVs recovered in bulk cultivation (Supplementary Fig. S4B). Regarding nutrient supplementation, 41% ASVs were found only in Pseud + SE cultivation and 12% were unique to Amx + SE cultivation. Thus, while the diversity of the community in droplets is statistically similar to bulk cultivation, these findings suggest that we can access different members of the groundwater microbiome (inoculum) in droplet cultivation, 6% of ASVs compared to 2% in bulk cultivation (Supplementary Fig. S4C). How this is reflected in the CPR part of the community was analysed next.

### Specific CPR taxa are enriched using droplet cultivation

**Figure 4:**
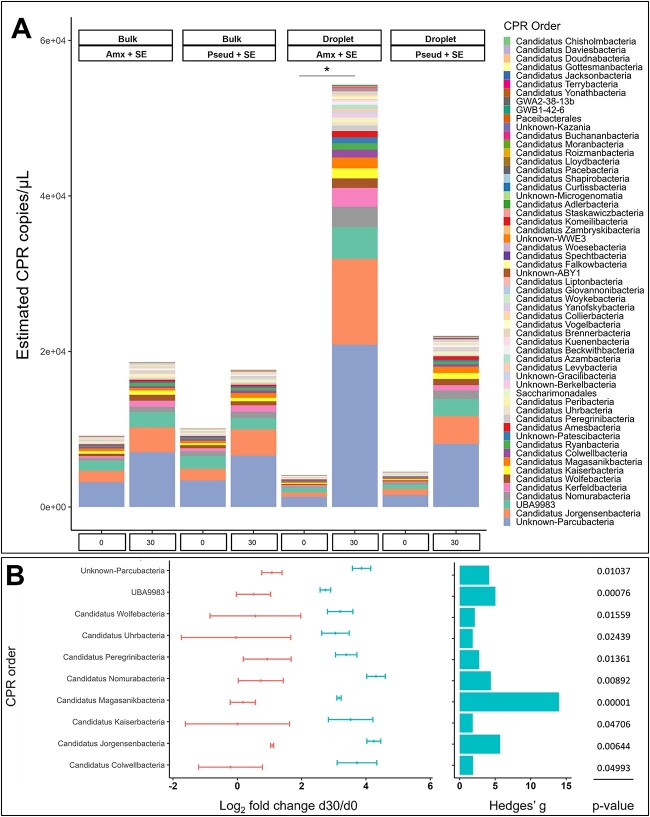
CPR enrichment at order-level in droplet and bulk cultivation. (A) Estimated abundance of CPR calculated via a combination of relative abundance data from 16S rRNA sequencing and quantification data from qPCR. Phylogenetic affiliation at the order level is shown for the sum of triplicates of each incubation condition. Significant difference is indicated between conditions (ANOVA-Tukey test, ^*^ (*P* < .05). (B) Change in estimated CPR abundance from day 0 to day 30 for the top 10 CPR orders of the Amx + SE sample. Bars depict the mean log_2_ fold change (±standard deviations) computed from estimated number of copies between day 30 and day 0 in three biological replicates using two cultivation methods: droplet (cyan) and bulk (coral). Displayed are the 10 most abundant orders of the total 57 assigned orders. Data normality was assessed by Shapiro–Wilk test, followed by Welch’s *t*-test for two independent samples comparison to generate corresponding *P*-values showing the differences between the cultivation methods in terms of fold change. Hedges’ *g*, indicating the effect size, was plotted as bars with colour corresponding to cultivation condition with the larger mean.

Relative abundance data from the amplicon sequencing and total 16S rRNA gene copy numbers based on qPCR were used to estimate quantitative CPR gene copy counts for each sample. While this conversion is biased by the use of different 16S primer sets optimized either for qPCR or sequencing, respectively, and does not acknowledge different 16S gene copy numbers, it allowed us to estimate the quantitative abundance of the different CPR, which we consider an important information for comparing the success of the different nutrition schemes and for the design of future-defined cultivation strategies. CPRs were enriched in all conditions after 30-day incubation with a reliable higher enrichment in droplet than bulk cultivation (Fig. 4A). The highest enrichment of CPR was observed in droplet cultivation with Amx + SE resulting in a significant 13.25-fold increase of CPR copies on day 30 as compared with day 0 (ANOVA-Tukey test *P* < .05), and almost 3-fold higher than bulk cultivation. Amx + SE droplets also displayed higher CPR Shannon diversity than bulk cultivation, while Pseud + SE showed comparable diversity in both cultivation methods (Supplementary Fig. S5A). The top 10 differentially enriched CPR orders with Amx + SE supplement inferred a significant difference between enrichment outcome of droplet and bulk cultures with an increase in estimated abundance of all these 10 CPR orders only for droplet cultivation (Fig. 4B). In contrast, most of these taxa were only occasionally sustained in bulk culture. None of the CPR orders were significantly enriched in the droplet-bulk cultivation comparison with the Pseud + SE supplement (Supplementary Fig. S5B). This shows that even in the superior droplet cultivation, a careful choice of the nutritional approach is crucial for an optimum or targeted CPR cultivation outcome.

### Coenrichment of non-CPR with CPR during droplet cultivation

**Figure 5:**
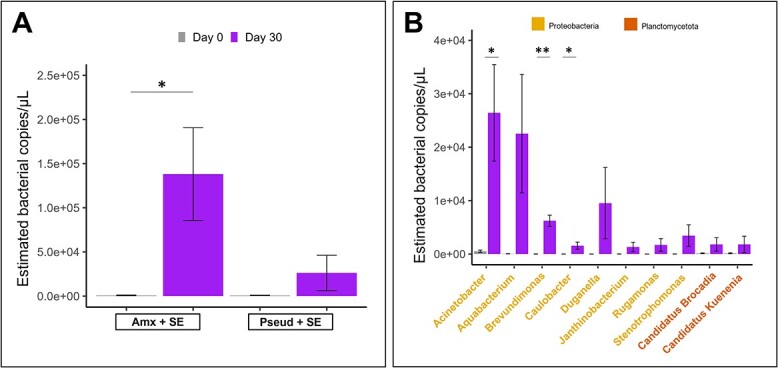
Quantification of the 11 most co-enriched non-CPR taxa. (A) Barplot depicts the mean and standard deviation of estimated number of bacterial 16S rRNA gene copies of genus Flavobacterium (phylum Bacteroidota) in Amx + SE and Pseud + SE at respective timepoints. (B) Barplot depicts the mean and standard deviation of the estimated number of gene copies of the other ten genera in Amx + SE at respective timepoints (corresponding data for the pseud + SE analysis are given in Supplementary Fig. S6B). Colour of the genera label represents their respective phylum. Samples were performed in triplicates; significant difference is indicated between conditions (unpaired *t* test, ^*^ [*P* < .05], ^*^^*^ [*P* < .01]).

Besides the CPR, non-CPR also thrived in the droplet cultivation (Supplementary Fig. S2C). We hypothesize that the droplets contained three main subpopulations: (i) containing only non-CPR cells, (ii) containing CPR with nonputative host cells, and (iii) CPR with putative host cells that actively supported CPR growth. To get further insights on putative hosts of the enriched CPR taxa, we analysed the progression of non-CPR taxa (582 ASVs after 30-day incubation; Supplementary Fig. S6A). The most coenriched non-CPR taxa at the genus level in the overall cultivation approach was the genus *Flavobacteria* (phylum Bacteroidota) in both Pseud + SE and Amx + SE on day 30 compared to day 0 (Fig. 5A). The following 10 enriched genera in Amx + SE were at least 1-fold lower than Flavobacteria, with 8 of them being members of the phylum Proteobacteria (Fig. 5B). Of those, the genera *Acinetobacter, Brevundimonas*, and *Caulobacter* were significantly increased. For Pseud + SE (Supplementary Fig. S6B), the 10 coenriched taxa were also dominated by the phylum Proteobacteria, accounting for seven genera, followed by three other genera from Bacteriodota, Nitrospirota (order Thermodesulfovibrionia), and Planctomycetota.

### Preliminary efforts for subcultivation of CPR in droplets

**Figure 6:**
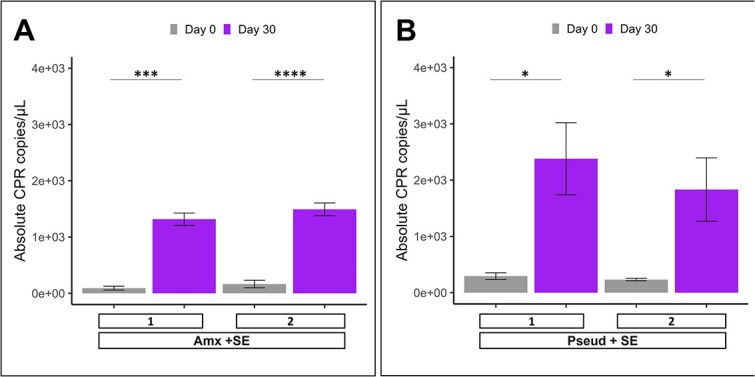
Quantification of CPR subculture subjected to different cultivation conditions in droplets. Barplots depict the mean and standard deviation of copy numbers of CPR quantified using a CPR-specific primer set with qPCR. Two droplet samples with enriched CPR were broken and re-encapsulated with fresh nutrient supplements (combination of soil extract and necromass—(A): Amx + SE and (B): Pseud + SE) on day 30 at λ_CPR_ = 5 followed by incubation for the subsequent 30 days. Samples were obtained at timepoint day 0 and day 30; CPR copies in corresponding sample were quantified. Samples were performed in triplicates; significant difference is indicated between conditions (unpaired *t* test, ^*^ [*P* < .05], ^*^^*^ [*P* < .01], ^*^^*^^*^ [*P* < .001], ^*^^*^^*^^*^ [*P* < .0001]).

To go one step further in obtaining a stable laboratory CPR coculture, we performed initial experiments to investigate the maintenance of enriched CPR by subcultivation in droplets. By breaking the pool of droplets on day 30 and re-encapsulating the bacteria at λ_CPR_ = 5 with fresh nutrients, the CPR population again enriched during 30 days of cultivation. The CPR copy number increased slightly for Amx + SE and was 10-fold lower than in the primary enriched culture for Pseud + SE (Fig. 6). The further sequencing attempt of pooled droplet samples at day 30 revealed no detectable CPR population. This is mainly due to the dominance of non-CPR bacteria in the pooled droplets after the first enrichment (Supplementary Fig. S2C). At a same load of CPR cells per droplets at the re-encapsulation, this led to an increment of total bacteria per droplet from λ = 12 in the first droplet culture to λ > 900 in the subculture. We therefore consider it as essential for our future work to develop a CPR marker to specifically detect droplets with enriched CPR. Then, CPR-containing droplets could be exclusively selected and sorted based on multiparametric analysis using our in-house optofluidic detection platform [58]. This will largely eliminate the bulk of droplets with the irrelevant non-CPR species, allowing more targeted re-encapsulation or even upscaling of individual droplets.

## Discussion

Here, we succeeded in performing sustainable laboratory enrichment of CPR from groundwater using a droplet ultrahigh-throughput cultivation method. This success was enabled by a combination of approaches: (i) successful harvest of CPR from groundwater, (ii) compartmentalization of small inocula into cultivation droplets, (iii) ultrahigh parallelization of cultivation to millions of cultures, and (iv) time-relevant qualitative assessment of CPR growth through a novel CPR-specific primer.

Using a low-volume filtration method followed by optimized cell detachment processes, we established a CPR harvesting protocol that preserves the abundance of groundwater CPR to generate a CPR-rich inoculum for cultivation. Initially, we evaluated different approaches for the harvest of CPR from the raw groundwater. Filter-size separation [59] was tested, and sequential filtration of groundwater on 0.2 and 0.1 μm filters was shown by others to retain both host-attached and free CPR [46]; however, it might risk the disruption of the often-fragile cell–cell attachment of symbiotic CPR. To reduce mechanical stress on microbial cells during the harvesting process, dead-end filtration was used to obtain the most viable and intact CPR, resulting in a 100× concentrated CPR inoculum for cultivation.

The inoculum was then applied in our advanced droplet microfluidic platform. At the rate of 1000 Hz, an average of five CPR cells were compartmentalized into a 150-pl droplet, allowing the long-term parallelized cultivation of 6.6 million stabilized monodispersed droplets in a total volume of 1 ml. Considering the importance of putative symbiosis of CPR taxa with other non-CPR hosts, including the host-associated replication behaviour [60], droplet cultivation allowed encapsulation of the slow-growing CPR with a small native microbial community. This approach might increase the probability for individual CPR to meet their putative hosts, while compartmentalization mitigates the risk of CPR being outcompeted. With this, we demonstrated the use of high-throughput cultivation to successfully enhance the enrichment of a wide range of CPR taxa. On the other hand, we acknowledge that the confined subset of droplets containing only non-CPR will need to be separated and removed before further subculturing of CPR-enriched communities becomes possible.

Monitoring the growth of this highly diverse superphylum requires sophisticatedly designed molecular tools. To date, standard universal 16S rRNA primers are the common tool in sequencing to investigate CPR taxa, but they tend to underestimate CPR populations due to the presence of introns in their 16S rRNA genes [61]. Other reported CPR primers are only target-specific towards members of Saccharibacteria [62, 63] and *Cand.* Absconditicoccus [30]. To dissect the highly abundant and intensely diverse CPR inhabited in the groundwater of the Hainich CZE, we designed a forward primer based on all aquatic CPRs available at the time of design. This primer targets 73% of all available Parcubacteria 16S rRNA sequences in the Silva database, with Parcubacteria being the most abundant CPR in groundwater samples. Pairing this primer with a universal reverse primer allowed the tracking of CPR growth directly in the lab, thereby efficiently and rapidly evaluating the most supportive cultivation conditions. In contrast, the 16S primer qPCR data were used to estimate quantitative CPR abundances from the 16S sequencing results. We acknowledge a potential coverage bias between these estimated CPR data from sequencing results and the direct CPR tracking during experiments because of the different use of primers.

To optimize the cultivation conditions, we added naturally occurring carbon and energy sources. Rainfalls lead to the vertical translocation of dissolved organic matter (DOM) and soil-derived microbial biomass into the subsurface, which may have a substantial impact on the dynamics of the groundwater microbiome [3, 42]. These complex carbon sources, such as DOM and bacterial cell lysate, have been shown to significantly enrich the diversity of groundwater [64]. By providing soil extract together with two different types of necromass, we demonstrated that the growth of CPR was significantly promoted, suggesting they or their host can thrive with the raw carbon substrates. In the campaign with Amx + SE and Pseud + SE supplements, CPRs were significantly enriched during the 30-day incubation in droplets but not in bulk culture (*P* < .0001). This exclusive enhancement is likely attributed to the predicted two distinct features offered by droplet cultivation: (i) confinement of CPR with a small number of native microbes that largely reduces competition and (ii) parallelization of millions of droplet chambers within a compact incubation that increases the probability of successful enrichment. Amplicon sequencing analysis provided additional evidence of the superior cultivation in droplets, especially demonstrated by the significant enrichment of several CPR orders in the Amx + SE condition. In contrast to Pseud necromass supplying nutrients from only one species, Amx necromass was derived from a range of Anammox species, including Planctomycetota, Bacteroidota, and Proteobacteria (Supplementary Fig. S3B), and offers a richer nutrient pool likely accommodating more diverse putative host taxa, resembling the effect upon groundwater recharge [43].

We assume that one of the major reasons for the success of CPR enrichment with our droplet cultivation lies in the availability of putative hosts. We attempted to further elucidate the non-CPR taxa pool originating from both CPR-containing droplets and CPR-free droplets to decipher potential CPR hosts based on their co-enrichment. The observed overall co-enrichment of Flavobacteria, Proteobacteria, and Planctomycetota agrees with earlier results of groundwater analysis from the Hainich CZE. In a metagenomic network analysis of groundwater samples, CPR were shown to interact with specific MAGs of the genus Prolixibacteraceae and Melioribacteraceae (phylum Bacteroidota) [34]. Moreover, based on time series data from the same site, the temporal co-occurrence pattern of CPR with members of the order Burkholderiales and the genus *Brocadia* was also reported [43]. Thus, repeatedly, these predominated non-CPR taxa are identified and appear to serve as potential hosts for supporting a symbiotic lifestyle of the enriched CPR. However, at this point, we cannot assess if they are co-enriched within the same droplets or in CPR-free droplets. To verify this, it will be essential to separate the droplet fraction that contains CPR from all those droplets in which non-CPR bacteria grew freely. Ongoing work is therefore investigating the design of specific optical detection probes for CPR to label and distinguish the CPR-containing droplets. With the information from this work, further strategies such as “emulsion, paired isolation and concatenation PCR” (epic-PCR) [65, 66] are currently being examined for the detection of interspecies interactions, which might open opportunities for a targeted co-isolation of CPR with defined hosts.

Overall, this method has shown great promise for enriching hard-to-culture bacteria and should also be investigated in other environments. The enrichments provide important information and insights for further downstream studies using microscopic techniques [21, 27], epic-PCR, and catalyzed reporter deposition-fluorescence in situ hybridization (CARD-FISH) [60, 67], to investigate the functional roles of uncultured microbes as well as host-associated interactions and finally to aid their controlled cultivation.

## Supplementary Material

supp_info_2nd_revision_V1_ycae080

Supp1-summed_Bactero_ASVs_ycae080

Supp2-summed_Proteo_ASVs_ycae080

Supp3-ASV_table_Relative_Abundance_ycae080

Supp4-taxa_table_ASVs_ycae080

Supp5-Metadata_16S_ycae080

Supp6-qPCR_data_new_ycae080

Supp7-Sample_label_Fig_S8_ycae080

Supp8-Read_count_16S_ycae080

## Data Availability

All raw and processed data for this manuscript are included in the supplementary information, and all amplicon sequence data were deposited to NCBI under the BioProject accession number PRJNA993466.

## References

[1] Castelle CJ , Banfield JF. Major new microbial groups expand diversity and alter our understanding of the tree of life. Cell 2018;172:1181–97. 10.1016/j.cell.2018.02.016 29522741

[2] Castelle CJ , Brown CT, Thomas BC et al. Unusual respiratory capacity and nitrogen metabolism in a Parcubacterium (OD1) of the candidate phyla radiation. Sci Rep 2017;7:40101. 10.1038/srep40101 28067254 PMC5220378

[3] Herrmann M , Wegner CE, Taubert M et al. Predominance of Cand. Patescibacteria in groundwater is caused by their preferential mobilization from soils and flourishing under oligotrophic conditions [Internet]. Front Microbiol 2019;10:1407. 10.3389/fmicb.2019.01407 31281301 PMC6596338

[4] Danczak RE , Johnston MD, Kenah C et al. Members of the candidate phyla radiation are functionally differentiated by carbon- and nitrogen-cycling capabilities. Microbiome 2017;5:112. 10.1186/s40168-017-0331-1 28865481 PMC5581439

[5] Hernsdorf AW , Amano Y, Miyakawa K et al. Potential for microbial H2 and metal transformations associated with novel bacteria and archaea in deep terrestrial subsurface sediments. ISME J 2017;11:1915–29. 10.1038/ismej.2017.39 28350393 PMC5520028

[6] Vigneron A , Cruaud P, Langlois V et al. Ultra-small and abundant: candidate phyla radiation bacteria are potential catalysts of carbon transformation in a thermokarst lake ecosystem. Limnol Oceanogr Lett 2020;5:212–20. 10.1002/lol2.10132

[7] Vavourakis CD , Andrei AS, Mehrshad M et al. A metagenomics roadmap to the uncultured genome diversity in hypersaline soda lake sediments. Microbiome 2018;6:1–18. 10.1186/s40168-018-0548-7 30231921 PMC6146748

[8] Moreira D , Zivanovic Y, López-Archilla AI et al. Reductive evolution and unique predatory mode in the CPR bacterium *Vampirococcus lugosii*. Nat Commun 2021;12:2454. 10.1038/s41467-021-22762-4 33911080 PMC8080830

[9] Probst AJ , Ladd B, Jarett JK et al. Differential depth distribution of microbial function and putative symbionts through sediment-hosted aquifers in the deep terrestrial subsurface. Nat Microbiol 2018;3:328–36. 10.1038/s41564-017-0098-y 29379208 PMC6792436

[10] Probst AJ , Castelle CJ, Singh A et al. Genomic resolution of a cold subsurface aquifer community provides metabolic insights for novel microbes adapted to high CO_2_ concentrations. Environ Microbiol 2017;19:459–74. 10.1111/1462-2920.13362 27112493

[11] Nicolas AM , Jaffe AL, Nuccio EE et al. Soil candidate phyla radiation bacteria encode components of aerobic metabolism and Co-occur with Nanoarchaea in the rare biosphere of rhizosphere grassland communities. mSystem*s* 2021;6:e0120520. 10.1128/mSystems.01205-20 34402646 PMC8407418

[12] Nascimento Lemos L , Manoharan L, William Mendes L et al. Metagenome assembled-genomes reveal similar functional profiles of CPR/Patescibacteria phyla in soils. Environ Microbiol Re*p* 2020;12:651–5. 10.1111/1758-2229.12880 32815317

[13] Lavy A , McGrath DG, Matheus Carnevali PB et al. Microbial communities across a hillslope-riparian transect shaped by proximity to the stream, groundwater table, and weathered bedrock. Ecol Evo*l* 2019;9:6869–900. 10.1002/ece3.5254 31380022 PMC6662431

[14] Tully BJ , Graham ED, JFH. The reconstruction of 2,631 draft metagenome-assembled genomes from the global oceans. Sci Dat*a* 2018;5:170203. 10.1038/sdata.2017.203 29337314 PMC5769542

[15] He X , McLean JS, Edlund A et al. Cultivation of a human-associated TM7 phylotype reveals a reduced genome and epibiotic parasitic lifestyle. Proc Natl Acad Sci US*A* 2015;112:244–9. 10.1073/pnas.1419038112 25535390 PMC4291631

[16] Bor B , Collins AJ, Murugkar PP et al. Insights obtained by culturing Saccharibacteria with their bacterial hosts. J Dent Re*s* 2020;99:685–94. 10.1177/0022034520905792 32075512 PMC7243422

[17] Murugkar PP , Collins AJ, Chen T et al. Isolation and cultivation of candidate phyla radiation Saccharibacteria (TM7) bacteria in coculture with bacterial hosts. J Oral Microbio*l* 2020;12:1814666. 10.1080/20002297.2020.1814666 PMC7651992 33209205

[18] Tsurumaki M , Saito M, Tomita M et al. Features of smaller ribosomes in candidate phyla radiation (CPR) bacteria revealed with a molecular evolutionary analysis. RN*A* 2022;28:1041–57. 10.1261/rna.079103.122 35688647 PMC9297842

[19] Cross KL , Campbell JH, Balachandran M et al. Targeted isolation and cultivation of uncultivated bacteria by reverse genomics. Nat Biotechno*l* 2019;37:1314–21. 10.1038/s41587-019-0260-6 31570900 PMC6858544

[20] Wrighton KC , Thomas BC, Sharon I et al. Fermentation, hydrogen, and sulfur metabolism in multiple uncultivated bacterial phyla. Science 2012;337:1661–5. 10.1126/science.1224041 23019650

[21] Luef B , Frischkorn KR, Wrighton KC et al. Diverse uncultivated ultra-small bacterial cells in groundwater. Nat Commu*n* 2015;6:6372. 10.1038/ncomms7372 25721682

[22] Beam JP , Becraft ED, Brown JM et al. Ancestral absence of electron transport chains in Patescibacteria and DPANN. Front Microbio*l* 2020;11:1–16. 33013724 10.3389/fmicb.2020.01848 PMC7507113

[23] Tian R , Ning D, He Z et al. Small and mighty: adaptation of superphylum Patescibacteria to groundwater environment drives their genome simplicity. Microbiom*e* 2020;8:1–15. 10.1186/s40168-020-00825-w 32252814 PMC7137472

[24] Kantor RS , Wrighton KC, Handley KM et al. Small genomes and sparse metabolisms of sediment-associated bacteria from four candidate phyla. MBi*o* 2013;4:1–11. 10.1128/mBio.00708-13 PMC3812714 24149512

[25] Rodríguez-Gijón A , Nuy JK, Mehrshad M et al. A genomic perspective across Earth’s microbiomes reveals that genome size in archaea and bacteria is linked to ecosystem type and trophic strategy. Front Microbio*l* 2022;12:1–9. 10.3389/fmicb.2021.761869 PMC8767057 35069467

[26] Wang Y , Hammes F, Boon N et al. Isolation and characterization of low nucleic acid (LNA)-content bacteria. ISME *J* 2009;3:889–902. 10.1038/ismej.2009.46 19421234

[27] Jaffe AL , Fuster M, Schoelmerich MC et al. Long-term incubation of lake water enables genomic sampling of consortia involving Planctomycetes and candidate phyla radiation bacteria. mSystems 2022;7:e00223–22. 10.1128/msystems.00223-22 PMC9040852 35353011

[28] He C , Keren R, Whittaker ML et al. Genome-resolved metagenomics reveals site-specific diversity of episymbiotic CPR bacteria and DPANN archaea in groundwater ecosystems. Nat Microbio*l* 2020;6:354–65. 10.1038/s41564-020-00840-5 PMC7906910 33495623

[29] Gong J , Qing Y, Guo X et al. “Candidatus Sonnebornia yantaiensis”, a member of candidate division OD1, as intracellular bacteria of the ciliated protist Paramecium bursaria (Ciliophora, Oligohymenophorea). Syst Appl Microbio*l* 2014;37:35–41. 10.1016/j.syapm.2013.08.007 24231291

[30] Yakimov MM , Merkel AY, Gaisin VA et al. Cultivation of a vampire: ‘CandidatusAbsconditicoccus praedator’. Environ Microbio*l* 2022;24:30–49. 10.1111/1462-2920.15823 34750952

[31] Anantharaman K , Brown CT, Hug LA et al. Thousands of microbial genomes shed light on interconnected biogeochemical processes in an aquifer system. Nat Commu*n* 2016;7:13219. 10.1038/ncomms13219 27774985 PMC5079060

[32] Brown CT , Olm MR, Thomas BC et al. Measurement of bacterial replication rates in microbial communities. Nat Biotechno*l* 2017;34:1256–63. 10.1038/nbt.3704 PMC5538567 27819664

[33] Wu X , Holmfeldt K, Hubalek V et al. Microbial metagenomes from three aquifers in the Fennoscandian shield terrestrial deep biosphere reveal metabolic partitioning among populations. ISME *J* 2016;10:1192–203. 10.1038/ismej.2015.185 26484735 PMC5029217

[34] Chaudhari NM , Overholt WA, Figueroa-Gonzalez PA et al. The economical lifestyle of CPR bacteria in groundwater allows little preference for environmental drivers. Environ Microbiom*e* 2021;16:1–18. 10.1186/s40793-021-00395-w 34906246 PMC8672522

[35] Mahler L , Niehs S, Martin K et al. Highly parallelized microfluidic droplet cultivation and prioritization on antibiotic producers from complex natural microbial communities. elif*e* 2021;10:64774. 10.7554/eLife.64774 PMC8081529 33764297

[36] Najah M , Calbrix R, Mahendra-Wijaya IP et al. Droplet-based microfluidics platform for ultra-high-throughput bioprospecting of cellulolytic microorganisms. Chem Bio*l* 2014;21:1722–32. 10.1016/j.chembiol.2014.10.020 25525991

[37] Zhou N , Sun YT, Chen DW et al. Harnessing microfluidic streak plate technique to investigate the gut microbiome of Reticulitermes chinensis. Microbiolog*y* 2019;8:e00654. 10.1002/mbo3.654 PMC6436436 29897677

[38] Villa MM , Bloom RJ, Silverman JD et al. Interindividual variation in dietary carbohydrate metabolism by gut bacteria revealed with droplet microfluidic culture. mSystems 2020;5:00864–19. 10.1128/msystems.00864-19 PMC7329328 32606031

[39] Watterson WJ , Tanyeri M, Watson AR et al. Droplet-based high-throughput cultivation for accurate screening of antibiotic resistant gut microbes. elif*e* 2020;9:1–22. 10.7554/eLife.56998 PMC7351490 32553109

[40] Hengoju S , Tovar M, Man DKW et al. Droplet Microfluidics for Microbial Biotechnolog*y*. Berlin, Heidelberg: Springer Berlin Heidelberg, 2020, 1–29.

[41] Küsel K , Totsche KU, Trumbore SE et al. How deep can surface signals Be traced in the critical zone? Merging biodiversity with biogeochemistry research in a central German Muschelkalk landscape [internet]. Front Earth Sc*i* 2016;4:32.

[42] Yan L , Herrmann M, Kampe B et al. Environmental selection shapes the formation of near-surface groundwater microbiomes. Water Re*s* 2020;170:115341. 10.1016/j.watres.2019.115341 31790889

[43] Yan L , Hermans SM, Totsche KU et al. Groundwater bacterial communities evolve over time in response to recharge. Water Re*s* 2021;201:117290. 10.1016/j.watres.2021.117290 34130083

[44] Chaudhary DK , Khulan A, Kim J. Development of a novel cultivation technique for uncultured soil bacteria. Sci Re*p* 2019;9:1–11. 31040339 10.1038/s41598-019-43182-x PMC6491550

[45] Tovar M , Weber T, Hengoju S et al. 3D-glass molds for facile production of complex droplet microfluidic chips. Biomicrofluidic*s* 2018;12:1–9. 10.1063/1.5013325 PMC5882410 29657658

[46] Barbara D , Hellemans J. The importance of quality control during qPCR data analysis. Int Drug Disco*v* 2010;18–31.

[47] Lou J , Yang L, Wang H et al. Assessing soil bacterial community and dynamics by integrated high-throughput absolute abundance quantification. Peer*J* 2018;6:e4514–9. 10.7717/peerj.4514 29576979 PMC5857175

[48] Jian C , Luukkonen P, Yki-Järvinen H et al. Quantitative PCR provides a simple and accessible method for quantitative microbiota profiling. PLoS On*e* 2020;15:1–10. 10.1371/journal.pone.0227285 PMC6961887 31940382

[49] Loy A , Lehner A, Lee N et al. Oligonucleotide microarray for 16S rRNA gene-based detection of all recognized lineages of sulfate-reducing prokaryotes in the environment. Appl Environ Microbio*l* 2002;68:5064–81. 10.1128/AEM.68.10.5064-5081.2002 12324358 PMC126405

[50] Daims H , Brühl A, Amann R et al. The domain-specific probe EUB338 is insufficient for the detection of all bacteria: development and evaluation of a more comprehensive probe set. Syst Appl Microbio*l* 1999;22:434–44. 10.1016/S0723-2020(99)80053-8 10553296

[51] Klindworth A , Pruesse E, Schweer T et al. Evaluation of general 16S ribosomal RNA gene PCR primers for classical and next-generation sequencing-based diversity studies. Nucleic Acids Re*s* 2013;41:e1–11. 10.1093/nar/gks808. 22933715 PMC3592464

[52] Lane DJ , Pace B, Olsen GJ et al. Rapid determination of 16S ribosomal RNA sequences for phylogenetic analyses (reverse transcriptase/dideoxynudeotide). Evolution (N Y*)* 1985;82:6955–9. 10.1073/pnas.82.20.6955 PMC391288 2413450

[53] Statistical, R Core Team . R: A Language and Environment Forcomputin*g*. Vienna A: R Foundation for Statistical Computing, 2021, U. No Title. www.R-project.org/

[54] Callahan BJ , McMurdie PJ, Rosen MJ et al. DADA2: high-resolution sample inference from Illumina amplicon data. Nat Method*s* 2016;13:581–3. 10.1038/nmeth.3869 27214047 PMC4927377

[55] Quast C , Pruesse E, Yilmaz P et al. The SILVA ribosomal RNA gene database project: improved data processing and web-based tools. Nucleic Acids Re*s* 2013;41:590–6. 10.1093/nar/gks1219 PMC3531112 23193283

[56] Oksanen J , Blanchet FG, Kindt R et al. Vegan: Community Ecology Package. R Package version 2.3. 2016.

[57] Hosokawa S , Kuroda K, Narihiro T et al. Cometabolism of the superphylum patescibacteria with anammox bacteria in a long-term freshwater anammox column reactor. Water 2021;13:208. 10.3390/w13020208

[58] Hengoju S , Wohlfeil S, Munser AS et al. Optofluidic detection setup for multi-parametric analysis of microbiological samples in droplets. Biomicrofluidic*s* 2020;14:1–12. 10.1063/1.5139603 PMC7148121 32547676

[59] Proctor CR , Besmer MD, Langenegger T et al. Phylogenetic clustering of small low nucleic acid-content bacteria across diverse freshwater ecosystems. ISME J 2018;12:1344–59. 10.1038/s41396-018-0070-8 29416124 PMC5932017

[60] Chiriac MC , Bulzu PA, Andrei AS et al. Ecogenomics sheds light on diverse lifestyle strategies in freshwater CPR. Microbiom*e* 2022;10:84–21. 10.1186/s40168-022-01274-3 35659305 PMC9166423

[61] Brown CT , Hug LA, Thomas BC et al. Unusual biology across a group comprising more than 15% of domain bacteria. Natur*e* 2015;523:208–11. 10.1038/nature14486 26083755

[62] Ibrahim A , Maatouk M, Rajaonison A et al. Adapted protocol for *Saccharibacteria* cocultivation: Two new members join the club of Candidate Phyla Radiation. Microbiol Spectr 2021;9:e01069–21. 10.1128/spectrum.01069-21 PMC8694215 35007432

[63] Takenaka R , Aoi Y, Ozaki N et al. Specificities and efficiencies of primers targeting Candidatus phylum Saccharibacteria in activated sludge. Materials (Basel*)* 2018;11:1129. 10.3390/ma11071129 PMC6073563 29970836

[64] Wu X , Spencer S, Gushgari-Doyle S et al. Culturing of Unculturable subsurface microbes: natural organic carbon source fuels the growth of diverse and distinct bacteria from groundwater. Front Microbiol 2020;11:610001. 10.3389/fmicb.2020.610001. PMC7773641 33391234

[65] Xie B , Wang J, Nie Y et al. Type IV pili trigger episymbiotic association of Saccharibacteria with its bacterial host. Proc Natl Acad Sc*i* 2022;119:e2215990119. 10.1073/pnas.2215990119 36454763 PMC9894109

[66] Spencer SJ , Tamminen MV, Preheim SP et al. Massively parallel sequencing of single cells by epicPCR links functional genes with phylogenetic markers. ISME J 2016;10:427–36. 10.1038/ismej.2015.124 26394010 PMC4737934

[67] Kubota K . CARD-FISH for environmental microorganisms: technical advancement and future applications. Microbes Enviro*n* 2013;28:3–12. 10.1264/jsme2.ME12107 23124765 PMC4070690

