# Supplementary material for: Enrichment of different taxa of the enigmatic candidate phyla radiation bacteria using a novel picolitre droplet technique
Source: ISME Commun. 2024 Jun 21;4(1):ycae080. doi: 10.1093/ismeco/ycae080 (PMC11214157; doi:10.1093/ismeco/ycae080)
Supplement: supp_info_2nd_revision_V1_ycae080 [file supp_info_2nd_revision_v1_ycae080.docx]

**Enrichment of different taxa of the enigmatic Candidate Phyla Radiation bacteria using a novel picolitre droplet technique**

*DeDe Kwun Wai Man^1,2^, Syrie M. Hermans ^2,3^, Martin Taubert^2,4^, Sarahi L. Garcia^5,6^, Sundar Hengoju^1^, Kirsten Küsel^2,4,7^, Miriam A. Rosenbaum^1,2,8*^*

1. Leibniz Institute for Natural Product Research and Infection Biology – Hans Knöll Institute - (HKI), Jena, Germany

2. Balance of the Microverse, Cluster of Excellence, Friedrich-Schiller-University, Jena, Germany

3. School of Science, Faculty of Health and Environmental Science, Auckland University of Technology, Auckland, Aotearoa New Zealand

4. Aquatic Geomicrobiology, Institute of Biodiversity, Faculty of Biological Sciences, Friedrich Schiller University, Jena, Germany

5. Department of Ecology, Environment and Plant Sciences, Science for Life Laboratory, Stockholm University, Stockholm, Sweden

6. Institute for Chemistry and Biology of the Marine Environment (ICBM), School of Mathematics and Science, Carl von Ossietzky Universität Oldenburg, 26129 Oldenburg, Germany

7. German Centre for Integrative Biodiversity Research (iDiv) Halle-Jena-Leipzig, Leipzig, Germany

8. Institute of Microbiology, Faculty of Biological Science, Friedrich Schiller University, Jena, Germany

*Corresponding author: Miriam A. Rosenbaum, [miriam.rosenbaum@leibniz-hki.de](mailto:miriam.rosenbaum@leibniz-hki.de)

**This section contains information for:**

[Additional materials and methods 3](#_Toc157703560)

[Nutrient supplements preparation 3](#_Toc157703561)

[Soil extract and Necromass sterility verification 3](#_Toc157703562)

[CPR-specific primer design and validation 4](#_Toc157703563)

[Establishment of CPR-specific primer-based qPCR 5](#_Toc157703564)

[Supplementary Figures 6](#_Toc157703565)

[Supplementary Table 12](#_Toc157703566)

[Supplementary Data 12](#_Toc157703567)

[Additional references 13](#_Toc157703568)

## Additional materials and methods

### Nutrient supplements preparation

Soil was collected from the” Schwellenburg” nature reserve, Germany (51° 1’ 51.9174” latitude, 10° 57’ 14.6013” longitude, 263 m altitude) in May 2016. After removing the surface turf, 12 kg of soil were collected until a depth of 10 cm. The soil was sealed in a plastic bag and transported back to the lab to process immediately. Soil was mixed 1:1 (w/w) with autoclaved distilled water, the mixture was then stirred for 2 h at 300 rpm at room temperature and left for sedimentation for another 2 h. Remaining insoluble particles in the decanted supernatant was removed by centrifugation at 15 970 xg for 20 min. The supernatant was then vacuum-filtered twice through sterilized cotton wool. Final filtration was performed with a Steriflip® filtration system (membrane pore size 0.22 µm) (Millipore, Germany) and aliquoted into 15-mL falcon tubes. All soil extracts were stored at -20°C.

Two types of necromass derived from a *Pseudomonas* sp. 002 culture isolated from Hainich CZE (1) and an anammox co-culture maintained planktonically in a semi-continuous stirred-tank reactor by the research group of Christian Jogler, FSU Jena were prepared. *Pseudomonas* sp. 002 were cultured for 24 h at 28°C in 10 mL of R2A medium. Anammox co-culture was collected freshly from the reactor. Cell counting was performed by hemocytometry and cultures were diluted to 10^11^ cells/mL. Cells were pelleted by centrifugation at 9 000 rpm, washed twice with autoclaved distilled water and resuspended in 1 mL of R2A medium. Sterile Ballotini glass beads at 0.25-0.5 mm were added to the cultures for homogenization using the FastPrep-24^TM^ (MP Biomedicals, US) for 5 x 1 min. Cell lysate was centrifuged at 13 000 rpm for 10 min, supernatant was then filtered with Steriflip® 0.22 µm filtration system. All necromass cell lysates were stored at -20°C. Soil extract and necromass were plated on NBE and M130 soil extract agar to ensure no trace of viable growth.

### Soil extract and Necromass sterility verification

Two agar media, NBE and Soil extract M130, were used for the plating of all nutrient supplements. 50 µL of each nutrient supplement was spread on agar plates and incubated at 28 °C for 7 days. No colony was observed in any of the plates. Viable DNA was detected in all the nutrient supplements which attributed to the ghost DNA detected in Illumina sequencing.

The agar recipes are as follows.

NBE:
1 g/L Bacto Beef extract (BD, Order Ref.Nr 212303), 2 g/L Bacto Yeast extract (BD Ref 212750), 5 g/L, Bacto peptone (BD Ref 211677), 5 g/L NaCl (Roth, ArtNr 9265, 15 g/L Agar (Roth, Art.Nr 2266), pH = 7,0

Soil extract agar M130:

150 g soil collected from the ”Schwellenburg” nature reserve were suspended in 600 mL distilled water, after stirring for 30 min soil particles were allowed to settle down, supernatant was decanted and passed through gauze and paper filter and centrifuged for 20 min at 14 000 rpm. The supernatant obtained was made up to 1 L with distilled water. Without pH regulation 20 g agar was added for solidification and sterilized for 35 min. at 121 °C. (Appearance: yellowish, clear, partly small, suspended particles)

### CPR-specific primer design and validation

Thirty-eight 16S rRNA sequences were downloaded from the [Integrated Microbial Genomes](https://en.wikipedia.org/wiki/Integrated_Microbial_Genomes_System) (Joint Genome Institute) CPR genomes available as July 2018.

>2264871603 16S rRNA. Bacterial SSU [candidate division ZB2 bacterium SCGC AAA255-P19 (SAK_001_138) : A255P19DRAFT_contig_2_0.4]

>2265015819 16S rRNA. Bacterial SSU [candidate division OD1 bacterium SCGC AAA011-N16 (Dusel_001_262) : A11N16DRAFT_contig_2_0.3]

>2265018473 16S rRNA. Bacterial SSU [candidate division OP11 bacterium SCGC AAA010-E09 (DUSEL_001_100) : A10E9DRAFT_contig_11_0.12]

>2609728233 16S rRNA. Bacterial SSU [Candidate division OD1 bacterium SCGC AD-602-F14 : Ga0070375_104]

>2619832517 16S rRNA. Bacterial SSU [Candidate division OD1 bacterium EBPR_Bin_1270 : Ga0073600_19]

>2623352529 16S rRNA. Bacterial SSU [Parcubacterium bin_V91210B2 Ga0074587 : Ga0074587_105]

>2623364956 16S rRNA. Bacterial SSU [Parcubacterium bin_V90308B3 Ga0074593 : Ga0074593_109]

>2623365800 16S rRNA. Bacterial SSU [Parcubacterium bin_V92810B11 Ga0074594 : Ga0074594_107]

>2623370384 16S rRNA. Bacterial SSU [Parcubacterium bin_V92810B4 Ga0074597 : Ga0074597_110]

>2648479513 16S rRNA. Bacterial SSU [Parcubacteria bacterium JGI MDM2 000213CP-K14 (contamination screened) : Ga0070038_106]

>2651939672 16S rRNA. Bacterial SSU [TM7 FNEB6 bin_26 : Ga0105423_109]

>2651960776 16S rRNA. Bacterial SSU [TM7 FNEF8-2 bin_14 : Ga0105436_103]

>2656364840 16S rRNA. Bacterial SSU [Parcubacteria bacterium DG_74_2 : Ga0111216_107]

>2656428798 16S rRNA. Bacterial SSU [Parcubacteria bacterium SG8_24 : Ga0111164_103]

>2657183012 16S rRNA. Bacterial SSU [Parcubacteria bacterium JGI MDM2 000213CP-M21 (contamination screened) : Ga0070032_103]

>2694851503 16S rRNA. Bacterial SSU [Parcubacteria bacterium JGI CrystG Apr02-2-C5 (unscreened) : Ga0098862_13]

>2694852060 16S rRNA. Bacterial SSU [Parcubacteria bacterium JGI CrystG Apr02-2-C5 (contamination screened) : Ga0098863_103]

>2694852834 16S rRNA. Bacterial SSU [Parcubacteria bacterium JGI CrystG Apr02-2-D16 (contamination screened) : Ga0098871_118]

>2694856385 16S rRNA. Bacterial SSU [Parcubacteria bacterium JGI CrystG Apr02-2-G4 (contamination screened) : Ga0098867_109]

>2694856886 16S rRNA. Bacterial SSU [Parcubacteria bacterium JGI CrystG Apr02-2-G4 (unscreened) : Ga0098866_107]

>2694862675 16S rRNA. Bacterial SSU [Parcubacteria bacterium JGI CrystG Apr02-2-I20 (contamination screened) : Ga0098909_133]

>2700388135 16S rRNA. Bacterial SSU [Parcubacteria bacterium JGI CrystG Apr02-3-B18 (contamination screened) : Ga0098915_112]

>2700499909 16S rRNA. Bacterial SSU [Parcubacteria bacterium JGI CrystG Apr02-2-N10 (contamination screened) : Ga0098877_115]

>2700504303 16S rRNA. Bacterial SSU [Parcubacteria bacterium JGI CrystG Apr02-3-D22 (contamination screened) : Ga0098879_108]

>2700504803 16S rRNA. Bacterial SSU [Parcubacteria bacterium JGI CrystG Apr02-3-J13 (contamination screened) : Ga0098883_101]

>2700505333 16S rRNA. Bacterial SSU [Parcubacteria bacterium JGI CrystG Apr02-3-J13 (unscreened) : Ga0098882_11]

>2700511560 16S rRNA. Bacterial SSU [Parcubacteria bacterium JGI CrystG Apr3-1-E17 (contamination screened) : Ga0098973_140]

>2700519228 16S rRNA. Bacterial SSU [Parcubacteria bacterium JGI CrystG Apr3-1-G16 (contamination screened) : Ga0099005_104]

>2700813536 16S rRNA. Bacterial SSU [Parcubacteria bacterium JGI CrystG Apr3-3-H12 (contamination screened) : Ga0099009_118]

>2700814217 16S rRNA. Bacterial SSU [Parcubacteria bacterium JGI CrystG Apr3-4-D20 (contamination screened) : Ga0098965_108]

>2700870125 16S rRNA. Bacterial SSU [Parcubacteria bacterium JGI CrystG Apr3-3-G16 (contamination screened) : Ga0099011_108]

>2700870766 16S rRNA. Bacterial SSU [Parcubacteria bacterium JGI CrystG Apr3-3-G16 (unscreened) : Ga0099010_108]

>2700873682 16S rRNA. Bacterial SSU [Parcubacteria bacterium JGI CrystG Apr3-1-E7 (contamination screened) : Ga0098961_118]

>2700914073 16S rRNA. Bacterial SSU [Parcubacteria bacterium JGI CrystG Aug02-3-N7 (contamination screened) : Ga0099107_11]

>2700920641 16S rRNA. Bacterial SSU [Parcubacteria bacterium JGI CrystG Aug02-3-N14 (contamination screened) : Ga0099131_109]

>2735958963 16S rRNA. Bacterial SSU [Parcubacteria bacterium JGI MDM2 ZSSED05-1-F14 (contamination screened) : Ga0191609_105]

>2736001173 16S rRNA. Bacterial SSU [Parcubacteria bacterium JGI MDM2 ZSSED05-1-D19 (contamination screened) : Ga0191633_102]

>2748078365 16S rRNA. Bacterial SSU [Parcubacteria bacterium JGI MDM2 ZSSED05-1-D20 (contamination screened) : Ga0191593_101]

One 16S rRNA sequence from an abundant freshwater microorganism was added as a negative reference.

>2506125100 16S rRNA. Bacterial SSU [actinobacterium SCGC AAA027-L06 (contigs) : A27L6_contig00019]

An alignment was performed using mothur v.1.33.3 using an aligned set of freshwater 16S rRNA genes database as template (Newton et al. 2011). Aligned sequences were further filtered. The filtered alignments were visualized using AliView and manually inspected to find a region common to all CPR that could be candidate for a primer. Once several regions were identified they were tested in TestProbe by arb-silva allowing no mismatches to check the specificity of the primer. The primer 684F- CPR-specific 3’ - GTAGKRRTRAAATSCGTT-5’ shows as of March 2024 to be targeting 0.26% of all bacterial 16S rRNA sequences, with 21% of the Patescibacteria 16S rRNA sequences, and less than 0.32% for Nitrospinota or any other bacterial Phyla and 2% of archaea. Further it targets 73% of all available Parcubacteria, 26% of ABY1, 22% of Berkelbacteria, 13% of Gracilibacteria, 6.3% of Kazania, 12% of Microgenomatia, 0.41% of Saccharimonadia, 14% of WWE3 and 25% of “unculutured” category in silva, proving its CPR coverage.

Amplicon sequencing for a subset of samples showed this primer set to be highly specific for CPR, with on average 97% of reads belonging to the CPR phylum ‘Patescibacteria’; the remaining reads were mostly unassigned (1.7%) or Proteobacteria (1.1%) (Supplementary Figure S8A). In this subset of samples, 17 different CPR orders were detected (Supplementary Figure S8B), indicating a diverse range of CPR present in the enrichments are covered by the primer set.

### Establishment of CPR-specific primer-based qPCR

#### Testing of primers with environmental sample

The CPR-specific primer 684F in combination with the universal bacterial primer 907R targeting 16S genes were tested using DNA obtained from groundwater samples of the Hainich Critical Zone Exploratory in August 2015 and February 2016. These samples are known to contain CPR in an average relative abundance of 30% (2). PCR was conducted in a 25 µL reaction using the HotStarTaq Master mix kit (Qiagen, Hilden, Germany) and the following conditions: initial denaturation at 95 °C for 15 minutes, 30 cycles of denaturation at 95 °C for 30 s, annealing at 52 °C for 30 s, elongation at 72 °C for 90 s, followed by final elongation at 72 °C for 10 min. The obtained PCR product was cloned into Escherichia coli JM109 chemically competent cells (Promega, Walldorf, Germany) using the pGEM-T Easy Vector system (Promega, Germany). Eleven clones were selected based on blue-white screening and the inserts were amplified using M13F/R primers as previously described (3), followed by Sanger Sequencing (Macrogen, Amsterdam, The Netherlands). Ten of the eleven inserts were classified as 16S rRNA gene fragments of different taxa of CPR, while one was related to the genus Gemmata.

To determine the specificity of the CPR-specific primers on groundwater samples, the obtained PCR product was subjected to amplicon sequencing. Library preparation was conducted using the NEBNext Ultra DNA Library Prep Kit (New England Biolabs GmbH, Frankfurt am Main, Germany), and sequencing was performed on an Illumina MiSeq platform (2 x 300 bp paired-end) using v3 chemistry. Bioinformatics analysis was done as described in the methods section. On average, more than 97% of sequence reads were affiliated with CPR.

#### Generation of qPCR standards

For an absolute quantification of CPR 16S rRNA gene copy numbers, four of the above-mentioned clones were used to generate a standard mix. Clones were selected based on the Sanger Sequencing results to cover a broad diversity of the detected CPR, including Cand. Nomurabacteria, Cand. Moranbacteria, Cand. Berkelbacteria and Cand. division WWE3. Plasmids were extracted from 2 mL of an overnight culture of the respective clones in LB medium using the GeneJET Plasmid Miniprep Kit (Thermo Fisher Scientific, Waltham, MA USA). DNA concentration of the plasmid extracts was determined with a NanoDrop spectrophotometer (Thermo Fisher Scientific), the four plasmids were mixed in an equimolar ratio and diluted to a final concentration of 10^8^ plasmids per µL.

## Supplementary Figures


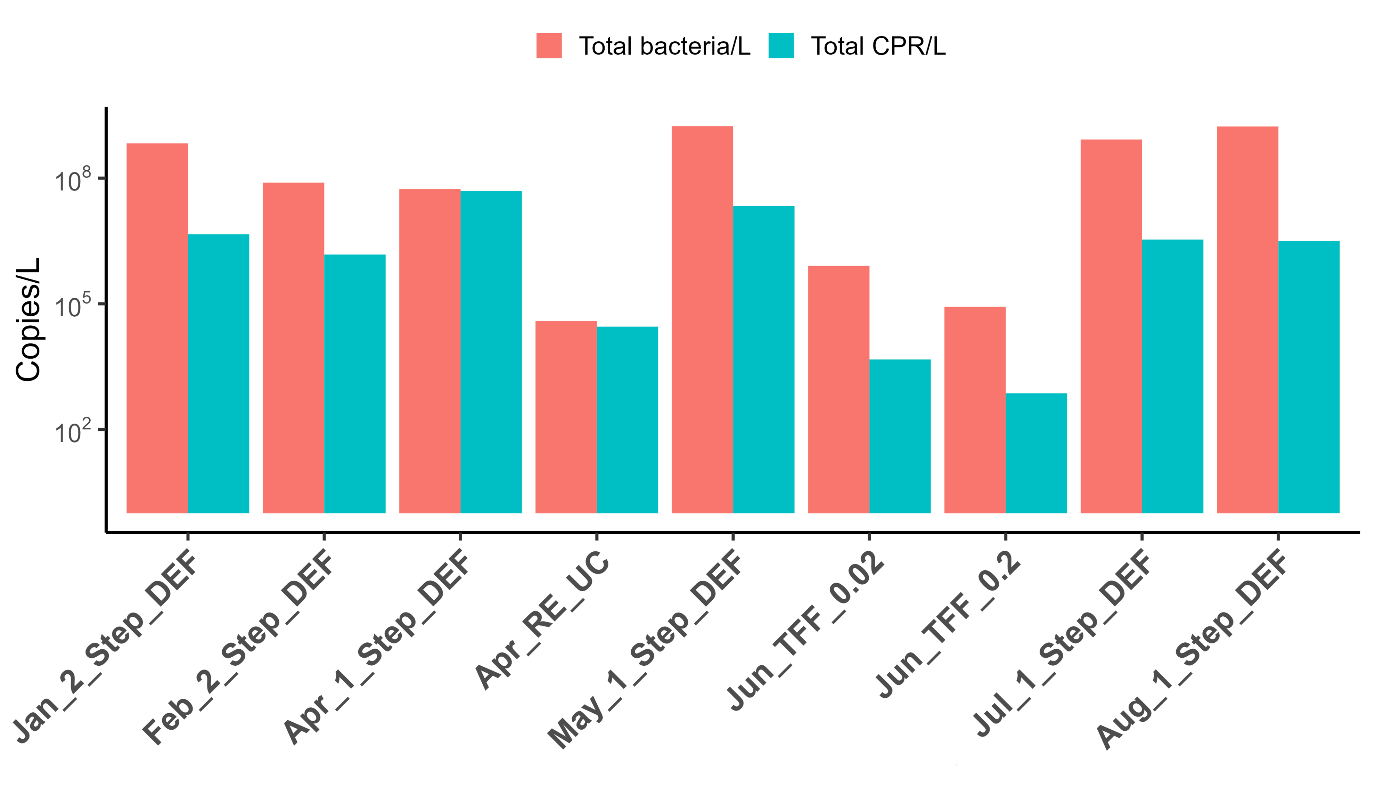


Supplementary Figure. S1. Bacterial harvest from consecutive batches of groundwater samples collected between January-August 2021 (6-week intervals) using different methods quantified with qCPR. Several cell-harvesting methods were performed including dead-end filtration (DEF) in 1-step (0.1 µm filter) or 2-step (sequential filtration on 0.45 µm followed by 0.1 µm filter), tangential flow filtration (TFF) using 0.2 µm or 0.02 µm cartridge and rotary evaporation followed by ultracentrifugation (RE-UC).


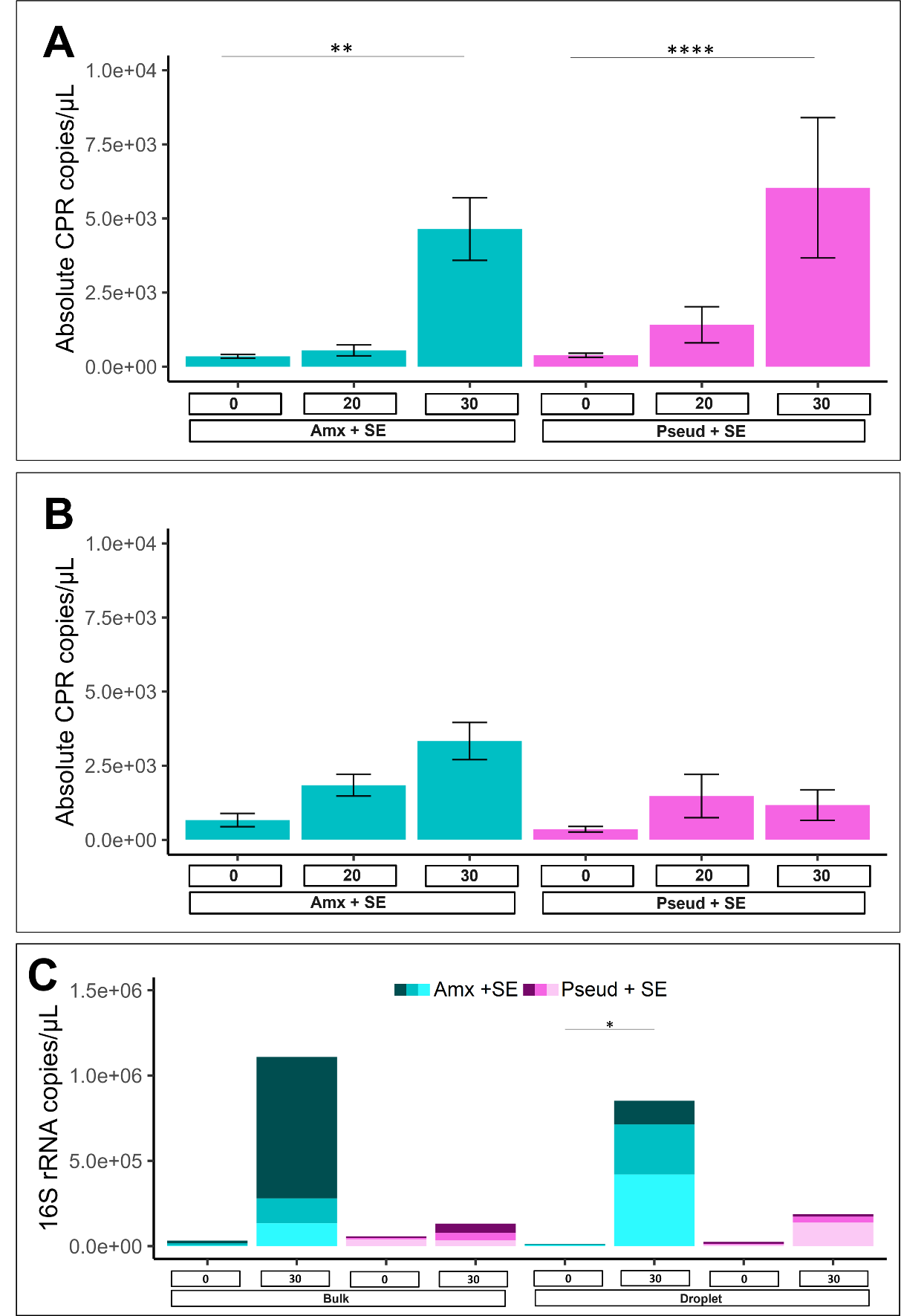


Supplementary Figure. S2. qPCR barplots with (A) CPR in droplet, (B) CPR in bulk and (C) total bacteria for both cultivation conditions. Barplots depict mean and standard deviations of copy numbers of (A/B): CPR quantified using ‘CPR-specific’ primer set with qPCR and (C) total bacteria quantified with the 16S primer set via qPCR. (A) and (B): Native groundwater microbial community in bulk cultivation with the addition of different nutrient supplement (a combination of soil extract and necromass (‘Amx + SE’/’Pseud + SE’)). Culture samples were obtained at timepoint Day 0, Day 20 and Day 30; CPR copies in corresponding sample were quantified. Significant difference is indicated between conditions. (ANOVA-Tukey test, ** (p < 0.01), **** (p < 0.0001)). (C) Total bacterial counts as determined via qPCR for the day 0 and day 30 samples of both cultivation conditions. The stacked bars show the sum of the total bacteria counts from the individual three replicates for each cultivation (we are not certain if the one data set from Bulk-Amx+SE, 30 days is really much higher in growth or if an unrepresentative sample, e.g. with cell clumps, was analysed). These data were used to calculate the estimated CPR abundance in Fig. 4 of the main manuscript.


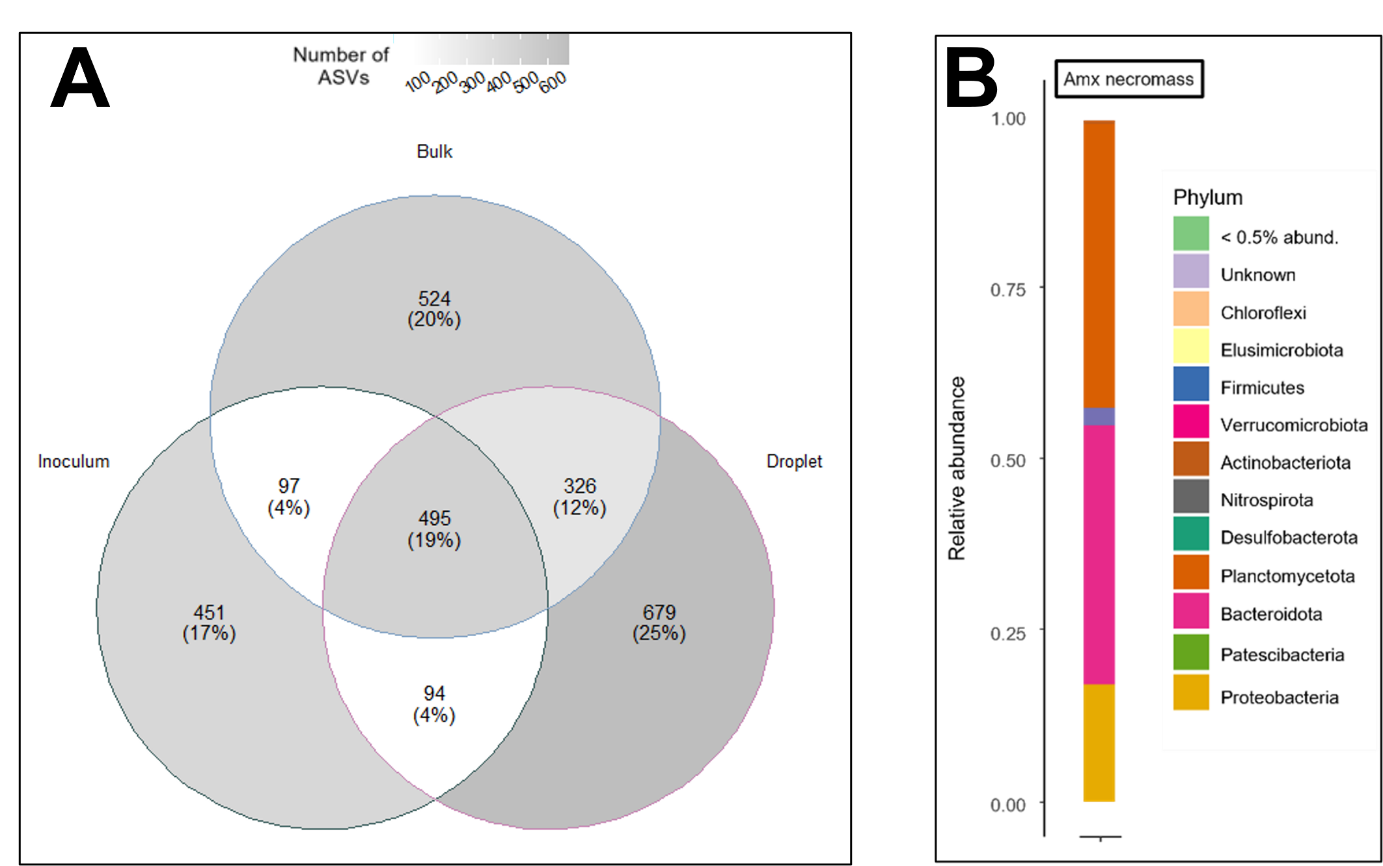


Supplementary Figure. S3. (A) Venn diagrams of ASVs in inoculum and all samples with different cultivation methods. (B) Community structure of Amx necromass based on 16S rRNA gene sequences (relative abundances). Phylogenetic affiliation at the phylum level is displayed.


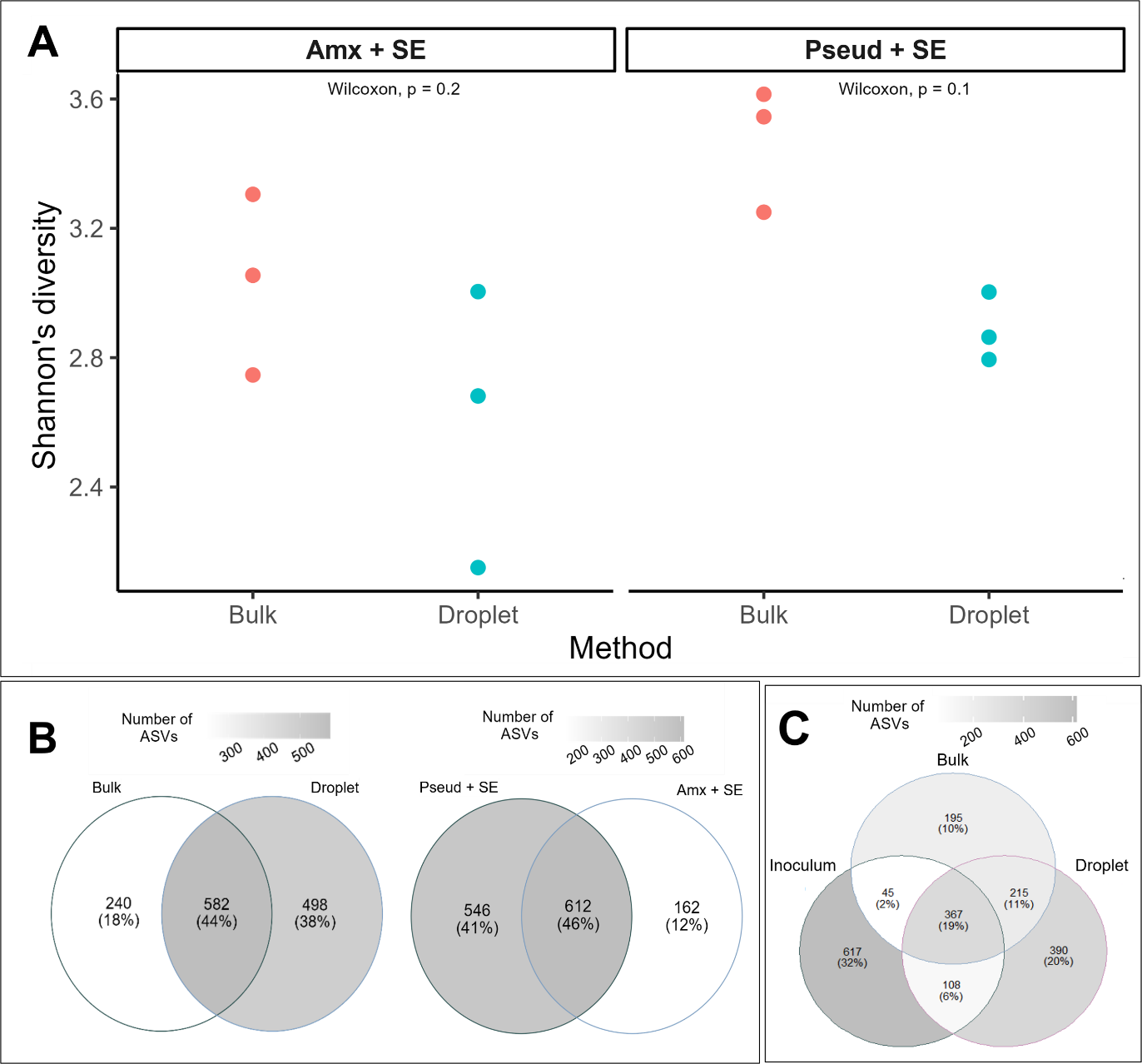


Supplementary Figure. S4. (A) Shannon diversity of day 30 samples of groundwater microbiome subjected to two cultivation conditions: droplet (cyan) and bulk (coral), with the addition of different nutrient supplement (a combination of soil extract and necromass (‘Amx + SE’/’Pseud + SE’)). Results from three replicate cultivations are shown, coloured dots represent method of cultivation in corresponding nutrient supplement condition. Wilcoxon tests were used to assess the differences between groups; p values are showed below the label of each condition. (B) Venn diagrams of cultivable ASVs on day 30 in different cultivation methods and nutrient conditions. (C) Venn diagrams of cultivable ASVs in inoculum and all day 30 samples with different cultivation methods.


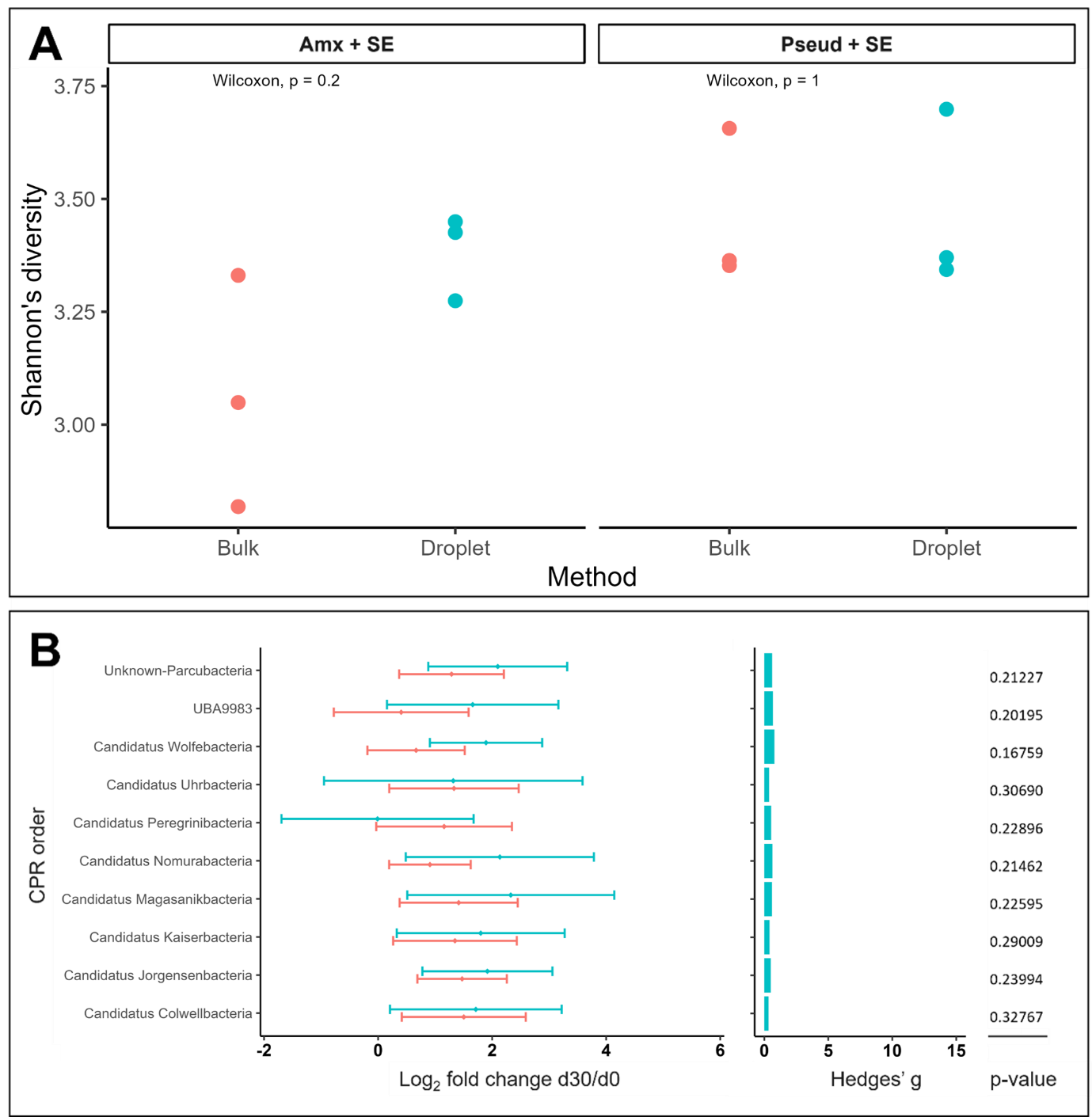


Supplementary Figure. S5. (A) Shannon diversity of CPR taxa of day 30 samples subjected to two cultivation conditions: droplet (cyan) and bulk (coral), with the addition of different nutrient supplement (a combination of soil extract and necromass (‘Amx + SE’/’Pseud + SE’)). Results from three replicates are shown, coloured dots represent method of cultivation in corresponding nutrient supplement condition. Wilcoxon tests were used to assess the differences between groups; p values are showed below the label of each condition. (B) Change in estimated CPR estimated abundance from day 0 to day 30 for ten CPR orders of the Pseud + SE sample. Bars depict the mean log_2_ fold change (± standard deviations) computed from estimated number of copies between day 30 and day 0 in three biological replicates using two cultivation methods. Displayed are ten of the most abundant orders of the total 57 assigned orders. Data normality was assessed by Shapiro-Wilk test, followed by Welch’s t-test for two independent samples comparison to generate corresponding p-values showing the differences between the cultivation methods in terms of fold change. Hedges’ g, indicating the effect size, was plotted as bars with colour corresponding to cultivation condition with the larger mean.


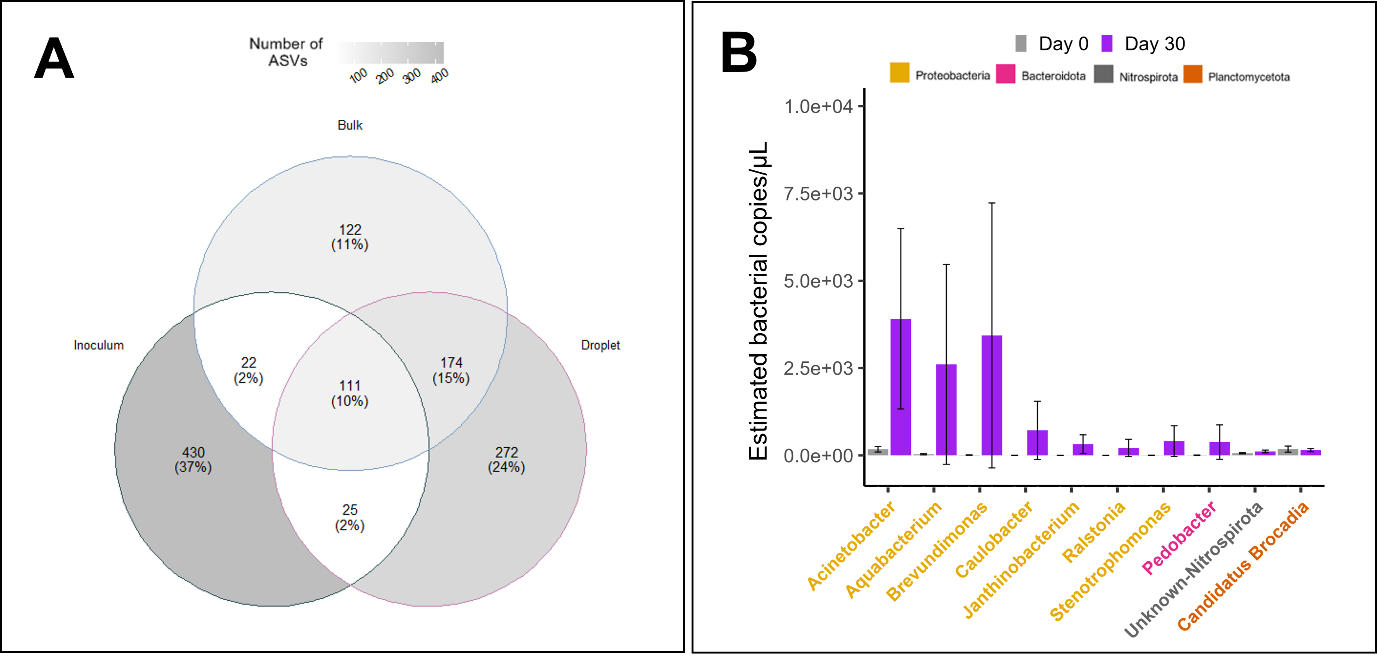


Supplementary Figure. S6. (A) Venn diagrams of cultivable non-CPR ASVs in inoculum and all day 30 samples with different cultivation methods. (B) Barplot depicts the mean and standard deviation of the estimated number of bacterial copies of the other ten genera (besides Flavobacterium – Figure. 5A) in Pseud + SE at respective timepoints. Color of the genera label represent their respective phylum. Samples were performed in triplicates. (Paired t test, n.s.)


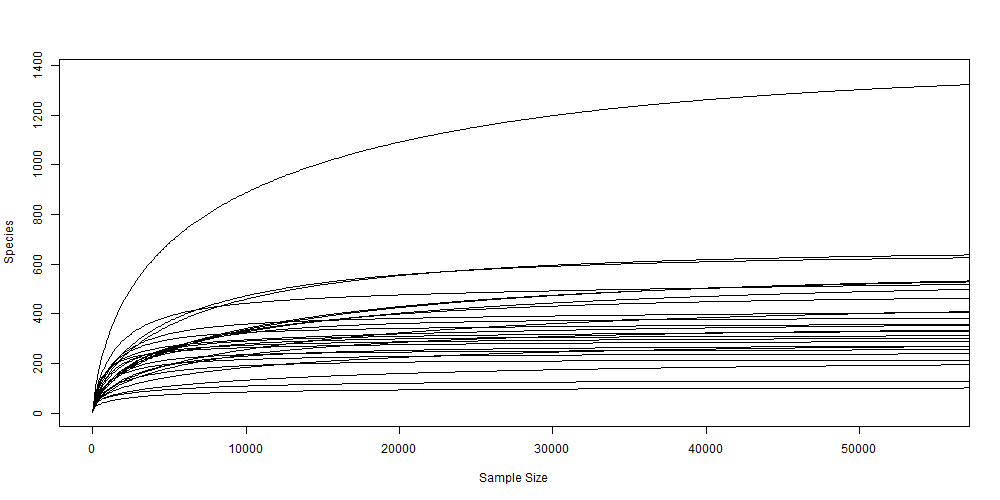


Supplementary Figure. S7. Rarefaction curves for all samples in this study. Samples were rarefied to 55 000 sequence reads. The top line represents the inoculum sample.


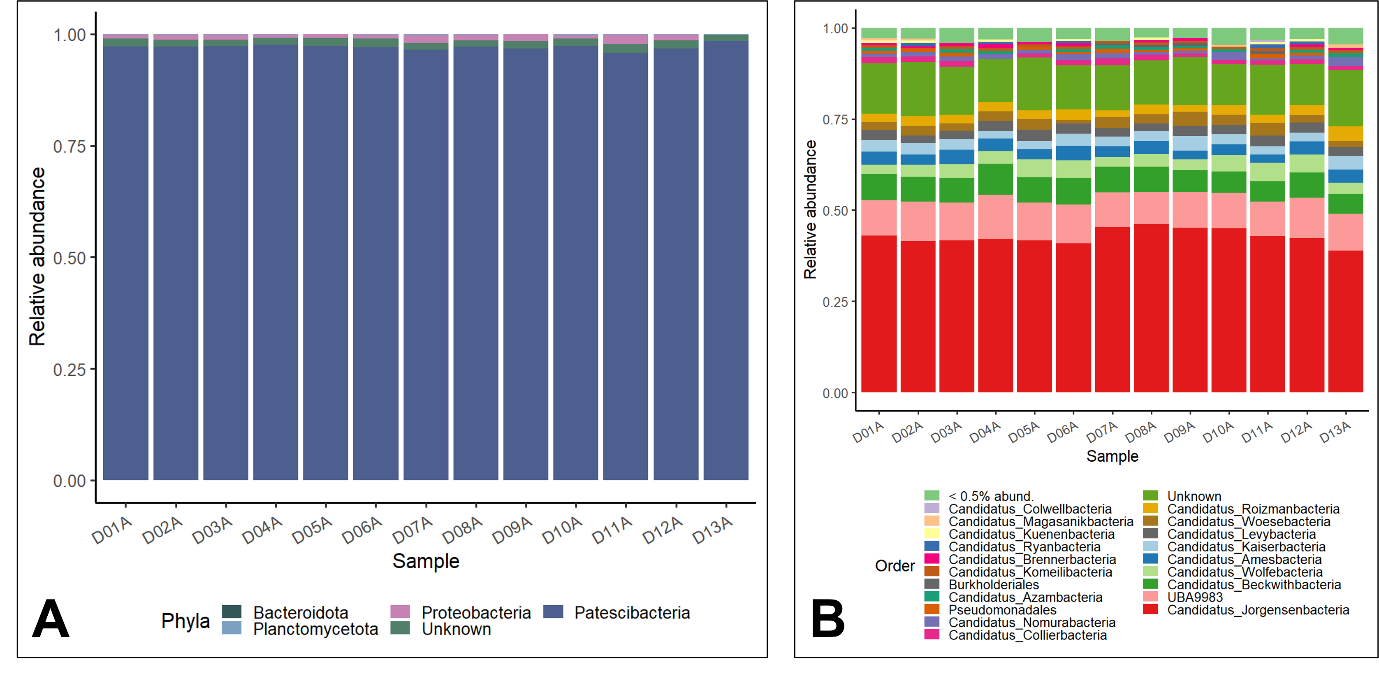


Supplementary Figure. S8. Amplicon sequencing on a subset of droplet cultivation samples using CPR-specific primer set. Relative abundance of community structure in each sample is displayed at phylum-level (A) and at order-level (B). Sample labels correspond to different cultivation conditions (Supplementary data 7).

## Supplementary Table

Supplementary Table S1: Results from the PERMANOVA. The following formula was used: formula BC~ method + nutrient + day, where BC is a Bray-Curtis matrix based on the ASVs assigned as Arthropoda at phylum level.

|  | **DF** | **SumsOfSqs** | **R2** | **F** | **Pr(>F)** |
| --- | --- | --- | --- | --- | --- |
| Method | 1 | 0.78 | 0.17 | 8.4 | 0.001 |
| Nutrient | 1 | 0.54 | 0.12 | 5.9 | 0.001 |
| Day | 1 | 1.39 | 0.31 | 15.1 | 0.001 |
| Residuals | 20 | 1.84 | 0.41 |  |  |

## Supplementary Data

1. Summed_bacteriotoda_ASVs
2. Summed_Proteo_ASVs
3. ASV_table_Relative_Abundance
4. taxa_table_ASVs
5. Metadata_16S
6. Sample_label_Fig_S8
7. qPCR_data
8. Read_count_16S

## Additional references

1. Geesink P, Taubert M, Jehmlich N, von Bergen M, Küsel K. Bacterial Necromass Is Rapidly Metabolized by Heterotrophic Bacteria and Supports Multiple Trophic Levels of the Groundwater Microbiome. Microbiol Spectr. 2022;10(4):1–14.

2. Herrmann M, Wegner CE, Taubert M, Geesink P, Lehmann K, Yan L, et al. Predominance of Cand. Patescibacteria in groundwater is caused by their preferential mobilization from soils and flourishing under oligotrophic conditions. Frontiers in Microbiology. 2019;10:1407.

3. Vielhauer GA, Fujino H, Regan JW. Cloning and localization of hFPS: A six-transmembrane mRNA splice variant of the human FP prostanoid receptor. Arch Biochem Biophys. 2004;421(2):175–85.
